# Antimicrobial resistance and genomic investigation of *Salmonella* isolated from retail foods in Guizhou, China

**DOI:** 10.3389/fmicb.2024.1345045

**Published:** 2024-03-06

**Authors:** Li Zhou, Qian Ye, Qian Zhou, Jian Wang, Guanqiao Li, Jingshu Xiang, Jingyu Huang, Yuanyuan Zhao, Tianli Zheng, Haojiang Zuo, Shijun Li

**Affiliations:** ^1^Guizhou Provincial Centre for Disease Control and Prevention, Guiyang, China; ^2^West China School of Public Health and West China Fourth Hospital, Sichuan University, Chengdu, China; ^3^Food Safety Monitoring and Risk Assessment Key Laboratory of Sichuan Province, Chengdu, China; ^4^Institute of Bioinformatics, Chongqing University of Posts and Telecommunications, Chongqing, China

**Keywords:** *Salmonella*, retail foods, Guizhou province, multi-drug resistance, whole genome sequencing

## Abstract

**Introduction:**

*Salmonella* is a major foodborne pathogen worldwide that causes severe morbidity and mortality. It is mainly caused by consuming contaminated food, with retail food considered the primary source.

**Methods:**

In Guizhou, China, 102 *Salmonella* strains isolated from 2016 to 2021 underwent phenotypic antimicrobial resistance testing and whole-genome sequencing (WGS) to understand Salmonella diversity, including serotypes, sequencing types (STs), antimicrobial genes, virulence genes, plasmid types, multi-locus sequence types (MLST), and core genome MLST (cgMLST).

**Results and discussion:**

*S.Typhimurium* was the dominant serotype, and O:4(B) was the leading serogroup. The most prevalent genotype was ST40. Phenotypic antimicrobial resistance identified 66.7% of the sampled isolates as multi-drug resistant (MDR). *S.Enteritidis* (*n* = 7), *S.Typhimurium* (*n* = 1), *S.Indiana* (*n* = 1), *S.*Kentucky (*n* = 1), *S.*Uganda (*n* = 1), all of which were MDR, were resistant to Colistin. Resistance rates varied significantly across different strains and food types, particularly meat products exhibiting higher resistance. Notably, significant increases in resistance were observed from 2016 to 2021 for the following: ≥ 1 resistant (*P* = 0.001), MDR (*P* = 0.001), ampicillin (*P* = 0.001), tetracycline (*P* < 0.001), chloramphenicol (*P* = 0.030), and trimethoprim/sulfamethoxazole (*P* = 0.003). The marked escalation in drug resistance over the recent years, coupled with the varying resistance rates among food sources, underscores the growing public health concern. Our findings highlight the need for a coordinated approach to effectively monitor and respond to Salmonella infections in Guizhou, China.

## 1 Introduction

Salmonella *spp*., highly prevalent foodborne pathogens, are responsible for a range of diseases from gastroenteritis to typhoid fever. They are mainly isolated from foods of animal origin, particularly meat, eggs, and their products (Liu et al., 2021; Alzahrani et al., 2023). Globally, approximately 200 million to over 1 billion cases of Salmonella occur each year, of which there are 93 million cases of gastroenteritis and 155,000 fatalities and 85% are linked to food consumption (Hung et al., 2017; Chlebicz and Slizewska, 2018; Castro-Vargas et al., 2020). In the United States, data on non-typhoidal Salmonella infections show an estimated annual occurrence of approximately 1.2 million cases, 23,000 hospitalizations, and 450 deaths (Scallan et al., 2011; Medalla et al., 2021). While in the European Union (EU), Salmonella caused the most outbreaks and outbreak-related illnesses. In 2021, 30 EU countries reported 60,494 laboratory-confirmed cases of salmonellosis, 73 of which were fatal. Compared with the number of cases in 2020, the number of cases in 2021 increased by 14% (European CDC, 2022). In China, an estimated 9.87 million cases of gastroenteritis are caused by Salmonella each year (Mao et al., 2011).

Guizhou Province is situated in the mountainous region of southwest China, spanning from 24°37′N to 29°130′N and 103°360′E to 109°350′E (Zuo et al., 2022; Xie and Zhang, 2023). It is known as a “natural encyclopedia” of the karst landform, has a population of approximately 38.56 million inhabitants, and covers an expansive area of around 1.76 × 105 km^2^. The unique geological features and landscapes of the region make it a popular tourist destination for exploring the beauty of karst formations. This province has recently experienced a boost in tourism owing to its abundant natural, cultural, and environmental resources. However, this increase in tourism may have also led to the spread of pathogens that cause diarrhea and antimicrobial resistance (Koch et al., 2011; Boolchandani et al., 2022). According to the “National Foodborne Disease Surveillance Work Manual” from China’s National Center for Food Safety Risk Assessment (Lu et al., 2023), *Salmonella* is the first-listed pathogen, along with *Vibrio parahaemolyticus, Diarrheagenic Escherichia coli, Shigella, and Norovirus*, as mandatory monitoring items for all provinces. This indicates the high priority given to *Salmonella* in the context of food safety and antimicrobial resistance. Our previous surveillance results have shown that food-related disease cases in Guizhou Province are mostly caused by *Salmonella typhimurium* (Wei et al., 2019; Bai et al., 2022; Long et al., 2022; Wei et al., 2023). Concerningly, we also have recently reported a foodborne outbreak in Guizhou caused by *Salmonella* (Zhou et al., 2023). Wide-ranging drug resistance and a high proportion of multi-drug resistant (MDR) strains have been isolated in meat, eggs, and their products. However, data on antimicrobial resistance and molecular genotyping of *Salmonella* spp. in Guizhou are lacking.

Recent advancements in genome analysis have significantly enhanced our understanding of *Salmonella*’s pathogenic mechanisms and epidemiology (Djordjevic et al., 2023). Genome sequencing and analysis provide insights into its genetic diversity, virulence factors, and antibiotic resistance patterns. This genomic information is crucial for developing effective therapies. Furthermore, comparative genomics among different *Salmonella* strains helps in tracing the source and transmission of outbreaks, thus playing a vital role in public health surveillance and response strategies (Kong et al., 2022; Fatima et al., 2023).

To better understand the characteristics of *Salmonella*, we characterized antimicrobial resistance, presence of antimicrobial resistance and virulence genes, and genetic characterization of *Salmonella* isolates in Guizhou between 2016 and 2021. These findings serve as valuable reference points for implementing effective measures for the prevention and control of *Salmonella* infections. Moreover, they promote the rational use of antimicrobial agents. By leveraging this information, appropriate strategies can be developed to mitigate the transmission of *Salmonella* and address the issue of antimicrobial resistance in the Guizhou Province.

## 2 Materials and methods

### 2.1 Strain source and collection

**Figure 1:**
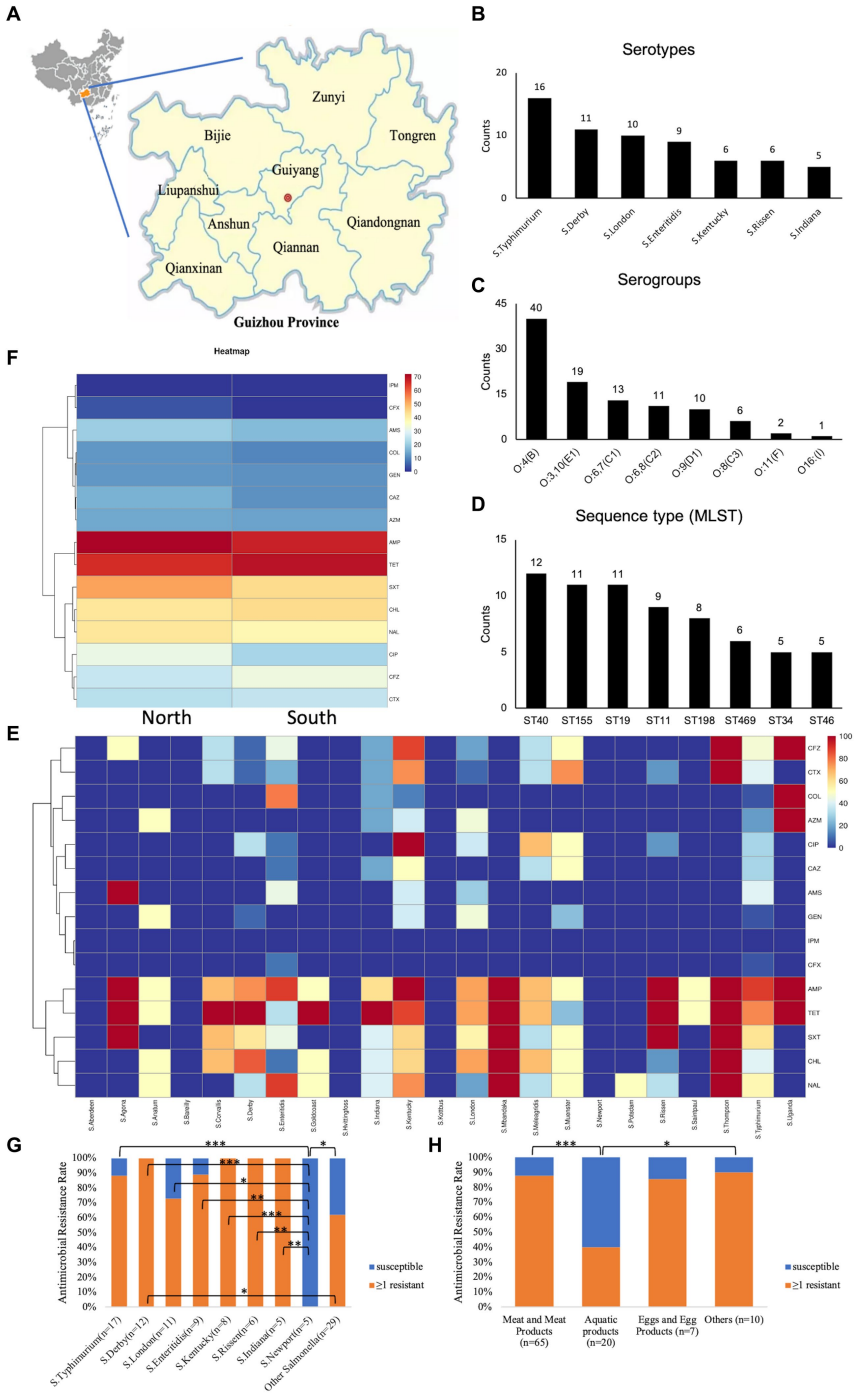
An overview of the isolated *Salmonella* strains’ classification and antimicrobial resistance patterns. **(A)** Source; **(B)** main serotypes; **(C)** serogroup; **(D)** primary sequence type via MLST; **(E)** antimicrobial resistance heatmap for diverse strains; **(F)** antimicrobial resistance heatmap by isolate region; **(G)** frequency of antibiotic resistance in 102 *Salmonella* isolates, Scale 0–100, representing 0–100% proportions; **(H)** *Salmonella* resistance distribution by food sources; ≥1 resistant, with at least one resistant strain; *p < 0.05; **p ≤ 0.01; ***p ≤ 0.001.

Strains were collected based on the Guizhou Foodborne Disease Surveillance Program. The study’s sampling period extended from 2016 to 2021. In this process, we methodically chose retail food items from all nine areas and prefectures of Guizhou Province (Figure 1A) to isolate *Salmonella*. The diverse assortment of food items sampled encompassed a range of categories, including meat and meat products (encompassing cooked meat products, raw poultry, raw livestock meat, and minced meat products), aquatic products, eggs and egg products, baked foods, soy products, rice and flour-based items (both in cooked and raw forms), various catering foods (like sushi, salads, and traditional Chinese cold dishes), as well as street vendors. In adherence to the project’s confidentiality protocols, specific details regarding the sample size and sampling plan are restricted internal information. Consequently, this study does not present the detection rate or other related findings.

### 2.2 *Salmonella* isolation and serotyping

*Salmonella* was isolated as previously described (GB4789.4-2016, 2016; Li et al., 2023). The suspected strains were subjected to CHROMagar *Salmonella* agar plates and a VITEK 2 compact automatic microbial identification system (BioMérieux, France). Positive colonies were subsequently confirmed by matrix-assisted laser desorption ionization time-of-flight mass spectrometry (MALDI-TOF-MS, Bruker Bremen, Germany). Single colonies obtained from the Swarm agar after overnight incubation at 37°C were selected for testing the phase 1 and phase 2 H antigens using slide agglutination. *Salmonella* antiserum Kits (60 V) were purchased from Statens Serum Institute (Copenhagen, Denmark) and Beijing Land Bridge Technology Ltd. (Beijing, China).

Based on the Guizhou Foodborne Disease Surveillance Program, from all retail food samples tested between 2016 and 2021, a total of 135 *Salmonella* strains were isolated. Due to limitations in detection capabilities and budget, a subset of 102 strains was subsequently selected for Antimicrobial Resistance (AMR) and Whole Genome Sequencing (WGS) analysis. Originally, the plan was to successfully sequence 100 of the 102 strains, acknowledging the possibility that 2 strains might fail in sequencing process. However, all 102 strains were successfully sequenced. Consequently, all 102 strains were utilized for the further antimicrobial susceptibility testing and whole genomic sequencing (Zhao et al., 2022; Petrin et al., 2023). Among these, 50 strains were from the northern region, and 52 were from the southern region. These strains were chosen for the following reasons: (1) complete coverage of all sampling geographic regions, (2) representation of positive isolates recovered from different retail foods, and (3) representation of positive isolates recovered from different years. Redundant strains with the same characteristics, such as the same serotype isolated from the same location and in the same year, were excluded. Given the selection criteria and our selection encompassed more than 75% (102/135) of the total samples, we believe that the chosen samples for this study are likely to be representative.

### 2.3 Antimicrobial susceptibility testing

The antimicrobial resistance of all *Salmonella* isolates was examined using a previously reported micro broth dilution method (Xingbai) (Kong et al., 2022). *Salmonella* isolates was tested against 15 antibiotics across 11 categories: ampicillin (AMP), ampicillin/sulbactam (AMS), tetracycline (TET), cefazolin (CFZ), cefotaxime (CTX), cephalosporin cefoxitin (CFX), ceftazidime (CAZ), imipenem (IMI), Gentamicin (GEN), Polymyxin E (Colistin, CT), Azithromycin (AZM), nalidixic acid (NAL), ciprofloxacin (CIP), chloramphenicol (CHL), and trimethoprim/sulfamethoxazole (SXT). MDR strains were defined as those resistance to three or more antimicrobial classes (Zhou et al., 2018, 2022).

Briefly, the operation was described as follows: A fresh, pure culture was taken and emulsified into a bacterial suspension with a turbidity equivalent to 0.5 McFarland standard in sterile water. Subsequently, 10 μL of this bacterial suspension was added to the Cation-Adjusted Mueller–Hinton Broth (CAMHB), which corresponded with the Thermo Sensititre^™^ Gram-negative drug susceptibility identification plate. In this broth, 50 μL of the bacterial broth solution was added to each microwell on the drug susceptibility plate. The solution was then incubated at 36°C for a duration of 18–24 h. Following incubation, the results of the drug susceptibility test were read using the Thermo Vizion automatic microbial susceptibility analysis system, in accordance with the guidelines provided by the Sensititre software. Blank and negative controls were also used. *Escherichia coli* ATCC 25922 and *Pseudomonas aeruginosa* ATCC 27853 were used as quality control strains to validate the results of the antimicrobial susceptibility testing (Kong et al., 2022). Antimicrobial breakpoints were determined in line with the interpretive standards provided by the Clinical Laboratory Standards Institute (CLSI) guidelines (CLSI, 2016).

### 2.4 Whole genome sequencing and bioinformatic analysis

The *Salmonella* strain was streaked onto Luria-Bertani (LB) plates. The plates were incubated overnight (approximately 18 h) at 37°C. Next, the single colony on the LB plate was inoculated into LB broth at 37°C under 150 rpm shaking conditions. Thereafter, *Salmonella* was harvested after 10 min centrifugation at 12,000 × g. Genomic DNA was extracted using Wizard^®^ Genomic DNA Purification Kit (Promega, Madison, Wisc, United States) according to the manufacturer’s protocol. Purified genomic DNA was quantified using the TBS-380 fluorometer (Turner BioSystems Inc., Sunnyvale, CA, United States). High-quality DNA (OD260/280 ≥ 1.5; ≥150 ng) was used for further analysis.

DNA samples were sheared into 400–500 bp fragments using a Covaris M220 Focused Acoustic Shearer following the manufacturer’s protocol. Illumina sequencing libraries were prepared from the sheared fragments using a Rapid DNA-Seq Kit (NEXTflex, San Jose, CA, United States). After end-repair, A-tailing, adapter ligation, and PCR enrichment, the libraries underwent paired-end sequencing (2 × 150 bp). Draft genome sequence analysis of the *Salmonella* strain was performed using an Illumina NovaSeq6000 sequencing platform.

Bioinformatic analysis of the Illumina data involved filtering raw reads with Fastp (version 0.19.6) (Chen et al., 2018), assembling with SOPA *de novo* version 2.04 (Luo et al., 2012), and predicting CDS, tRNA, and rRNA with Glimmer, tRNA-scan-SE, and Barrnap, respectively (Xu et al., 2023). Annotations were derived from NR, Swiss-Prot, Pfam, GO, COG, and KEGG databases using tools like BLASTP, Diamond, and HMMER. Briefly, each set of query proteins was aligned with the databases, and annotations of the best-matched subjects (*e*-value < 10^−5^) were obtained for gene annotation. Based on the Comprehensive Antibiotic Resistance Database (CARD) and the Virulence Factor Database (VFDB), the antibiotic resistance and virulence genes for each *Salmonella* isolate were evaluated (Akter et al., 2023). The Plasmid replicons for the genome sequences were studied using PlasmidFinder (Akter et al., 2023). The sequencing results for *Salmonella* strains were uploaded to the National Foodborne Disease Molecular Tracing Network (TraNet). Multi-locus sequence typing (MLST) and core genome MLST (cgMLST) analysis were performed using BioNumerics version 7.6 software (Applied Maths, Sint-Martens-Latem, Belgium) (Kong et al., 2022). Complete linkage was used to construct a cluster analysis dendrogram. For these analyses, default software parameters were consistently utilized.

### 2.5 Statistical analysis

All reported data were audited, checked, exported, and managed using Microsoft Excel 2016. Variable values are reported either as counts or percentages (%) for categorical data, or as mean ± standard deviation for continuous data. Statistical analyses of resistance rates and MDR profiles were conducted using R version 4.1.2 (R Foundation for Statistical Computing, Vienna, Austria) or SPSS version 25 (IBM Corp, Armonk, NY, United States). Besides, stratified analyses (Mukherjee et al., 2019; Marchello et al., 2022) by year were also conducted. Based on the characteristics of the data, appropriate statistical tests were selected for analysis, including the Chi-square test/Fisher’s test for categorical data and the *t*-test/Mann–Whitney U test for continuous data. The Mantel–Haenszel χ^2^ test was employed to assess trends. A *p*-value < 0.05 was deemed statistically significant.

## 3 Results

### 3.1 Distribution of *Salmonella* isolates

From 2016 to 2021, 102 strains of *Salmonella* were isolated from retail foods in nine distinct regions of Guizhou Province: Bijie, Zunyi, Tongren, Liupanshui, Anshun, Guiyang, Qianxinan (Miao and Dong autonomous prefectures in southeast Guizhou), Qiannan (Buyi and Miao autonomous prefectures in south Guizhou), and Qiandongnan (Buyi and Miao autonomous prefectures in southwest Guizhou, Figure 1A).

Table 1 shows the distribution of *Salmonella* isolates among food types and sampling sites. In total, 102 *Salmonella* isolates were obtained. No significant differences were observed between the sampling sites in northern and southern Guizhou. However, considering food types, *Salmonella* was more common in meat and meat Products (63.70%; 65/102) than in aquatic products 19.60% (20/102) and “other origin” samples 9.80% (10/102).

**Table 1:** Distribution of *Salmonella* isolates among food types and sampling sites.

Food types	North Guizhou	South Guizhou	Total
Meat and meat products	70.00% (35/50)	57.69% (30/52)	63.70% (65/102)
Aquatic products	14.00% (7/50)	25.00% (13/52)	19.60% (20/102)
Eggs and egg products	4.00% (2/50)	9.62% (5/52)	6.90% (7/102)
Others	12.00% (6/50)	7.69% (4/52)	9.80% (10/102)
Total	49.02% (50/102)	50.98% (52/102)	100% (102/102)

The slide agglutination test revealed the presence of 32 different serotypes and eight serogroups among the 102 studied *Salmonella* strains. The dominant serotype was *Typhimurium* (15.7%; 16/102, Figure 1B), the dominant serogroup was O:4 (B) (39.2%; 40/102, Figure 1C), and among the *Salmonella* strains under investigation, sequence type ST40 was the most prevalent (Figure 1D). Moreover, approximately one-third of agglutination-based serotypes did not match the MSLT-based serotypes (Supplementary Data Sheet 1_“AST” sheet). To enhance accuracy and reduce potential human errors in serotype determination from hemagglutination tests, which could lead to misjudgment, our subsequent analysis focused on analyzing predicted serotypes derived from sequencing types.

According to Supplementary Figure S1, in meat, the main *Salmonella* strains are *S.* London, *S.* Derby, *S.* Kentucky, *S. enteritidis*, *S.* Rissen, and *S. typhimurium*. In aquatic food, the main strains are *S.* Newport and *S. typhimurium*. In eggs and other food, the main strains are *S.* London and *S. typhimurium*, respectively.

### 3.2 Phenotypic antimicrobial resistance

Table 2 shows that the highest resistance was observed for ampicillin (69.6%; 71/102), followed by tetracycline (67.7%; 69/102) and trimethoprim/sulfamethoxazole (48.0%; 49/102), whereas no instance of resistance was observed for imipenem (0.0%; 0/102). Of the isolated strains, 78.4% (80/102) of the isolated strains were resistant to at least one antimicrobial class, 72.5% (74/102) were resistant to at least two antimicrobial classes, and 66.7% (68/102) were resistant to three or more than three antimicrobial classes, namely MDR strains. Moreover, our results showed that 16.7% (17/102) of isolates were resistant to eight antimicrobial classes, 10.8% (11/102) were resistant to nine antimicrobial classes, and no isolates (0/102) were resistant to all tested antimicrobial agents (Supplementary Data Sheet 1_“AST” sheet).

**Table 2:** Drug resistance patterns of *Salmonella* isolates.

Antimicrobial classes	Antimicrobial agents	Abbreviations	Resistant criteria (μg/mL)	Resistant rate (%)
Penicillin	Ampicillin	AMP	≥32	69.61% (71/102)
Tetracyclines	Tetracycline	TET	≥16	67.65% (69/102)
Folate pathway inhibitors	Trimethoprim/Sulfamethoxazole	SXT	≥4/76	48.04% (49/102)
Phenylpropanol	Chloramphenicol	CHL	≥32	43.14% (44/102)
Quinolones and fluoroquinolones	Nalidixicacid	NAL	≥32	40.20% (41/102)
	Ciprofloxacin	CIP	≥1	26.47% (27/102)
Cephalosporins	Cefazolin	CFZ	≥8	29.41% (30/102)
	Cefotaxime	CTX	≥4	24.51% (25/102)
	Ceftazidime	CAZ	≥16	13.73% (14/102)
	Cefoxitin	CFX	≥32	1.96% (2/102)
β-lactams combination	Ampicillin/Sulbactam	AMS	≥32/16	18.63% (19/102)
Macrolide	Azithromycin	AZM	≥32	13.73% (14/102)
Aminoglycosides	Gentamicin	GEN	≥16	11.76% (12/102)
Lipopeptide	Colistin	COL	≥4	10.78% (11/102)
Carbapenems	Imipenem	IPM	≥4	0.00% (0/102)

The distribution of antimicrobial resistance showed that some *S. Kentucky* (16.7%, 1/6) and *S. typhimurium* (11.8%, 2/17) isolates were the most resistant (resistant to ≥11 antibiotics), while *S.* Aberdeen (*n* = 1), *S.* Bareilly (*n* = 1), *S.* Hvittingfoss (*n* = 1), *S.* Kottbus (*n* = 1), and *S. Newport* (*n* = 5) were sensitive to all tested antimicrobial agents (Figure 1E). *S. enteritidis* (*n* = 7), *S. typhimurium* (*n* = 1), *S. Indiana* (*n* = 1), *S.* Kentucky (*n* = 1), and *S.* Uganda (*n* = 1), all of which were MDR, were resistant to colistin (Supplementary Data Sheet 1_“AST” sheet). Concerning the impact of the sampling location on the antimicrobial resistance of isolates, our findings demonstrated that the *Salmonella* strains obtained from North Guizhou displayed similar resistance levels to those from South Guizhou (Figure 1F, *p* > 0.05). In the stratified analysis of the major *Salmonella* strains (n ≥ 5), it was observed that except for *S.* Newport, which had a 0% drug resistant rate, the resistance rates of the other strains were all ≥70% (Figure 1G). Specifically, the resistance rate in *S.* Newport was significantly lower compared to *S.* Kentucky, *S. typhimurium*, those with isolates ≥5, and the other *Salmonella* strains with fewer than 5 isolates (*p* < 0.05, Figure 1G). In contrast, all 12 *S.* Derby strains exhibited 100% resistance, significantly higher than other *Salmonella* strains with fewer than 5 isolates (*p* < 0.05, Figure 1G). In the stratified analysis by food sources, the resistance rate of *Salmonella* isolated from aquatic products (40.0%) was significantly lower than that from meat (87.7%) and other sources (90.0%, *p* < 0.05, Figure 1H).

**Figure 2:**
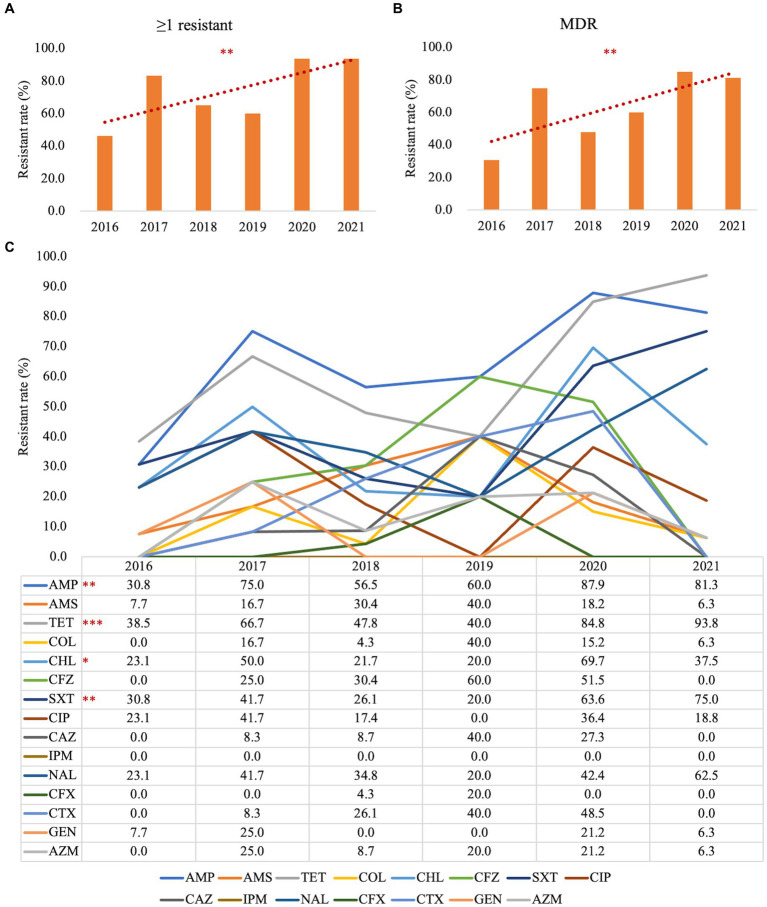
Stratified analysis by year for antimicrobial susceptibility testing in *Salmonella*. **(A)** Trends in the *Salmonella* antimicrobial resistance rate with ≥1 resistant from 2016 to 2021; **(B)** Trends in *Salmonella* antimicrobial resistance rate for MDR from 2016 to 2021; **(C)** Trends in *Salmonella* antimicrobial resistance rate for each antimicrobial reagent from 2016 to 2021; ≥1 resistant, with at least one resistant strain; MDR, multi-drug resistant; AMP, ampicillin; AMS, ampicillin/sulbactam; TET, tetracycline; CFZ, cefazolin; CTX, cefotaxime; CFX, cephalosporin cefoxitin; CAZ, ceftazidime; IMI, imipenem; GEN, gentamicin; CT, polymyxin E; AZM, azithromycin; NAL, nalidixic acid; CIP, ciprofloxacin; CHL, chloramphenicol; SXT, trimethoprim/sulfamethoxazole. *p < 0.05; **p ≤ 0.01; ***p ≤ 0.001; *n* = 102.

To address the trends in antimicrobial resistance in retail foods from 2016 to 2021, we evaluated the yearly changes in resistance. Significant increases in resistance were observed from 2016 to 2021 for the following: ≥1 resistant (Figure 2A, *p* = 0.001), MDR (Figure 2B, *p* = 0.001), AMP (Figure 2C, *p* = 0.001), TET (Figure 2C, *p* < 0.001), CHL (Figure 2C, *p* = 0.030), and SXT (Figure 2C, *p* = 0.003). However, no significant increase in resistance rates was detected for the other drugs examined. Due to the limited sample size in different retail food types, it was not feasible to stratify these trends further by each category.

### 3.3 Genotypic antimicrobial resistance

**Figure 3:**
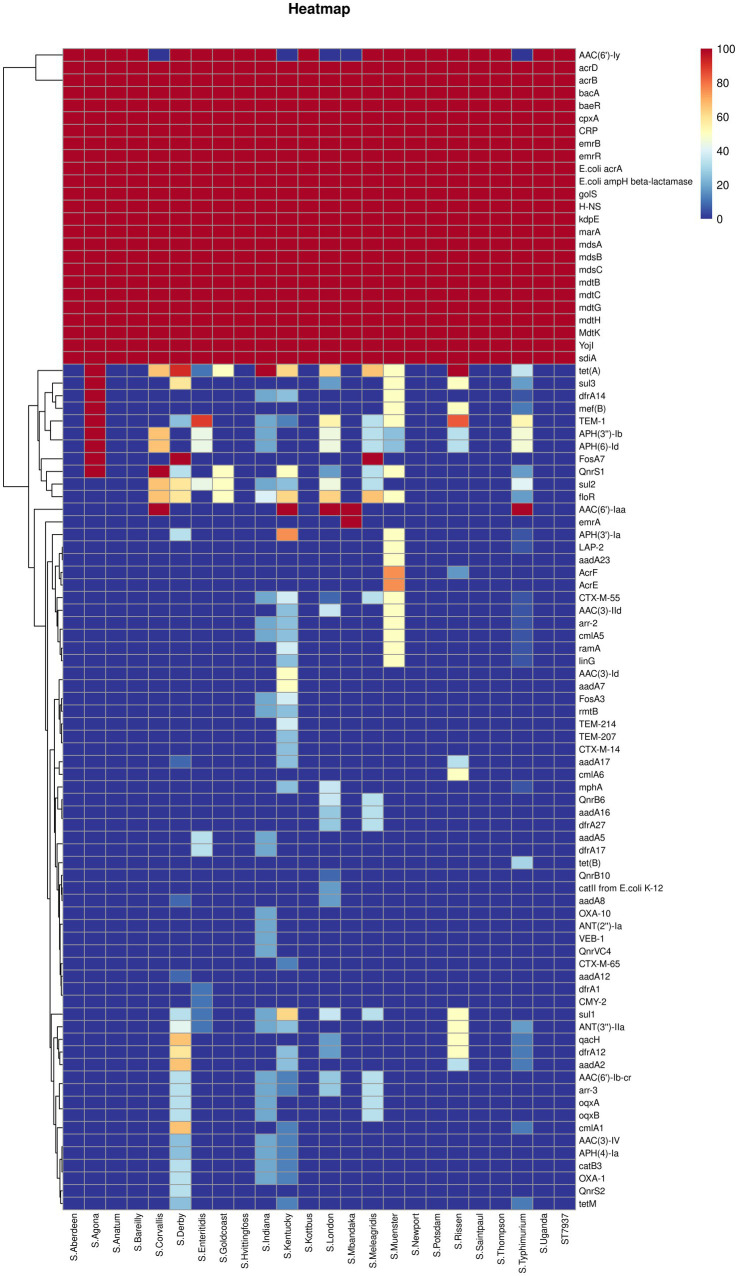
The heatmap of antimicrobial resistance genes in the studied *Salmonella* strains. Scale 0–100, representing 0–100% proportions.

**Figure 4:**
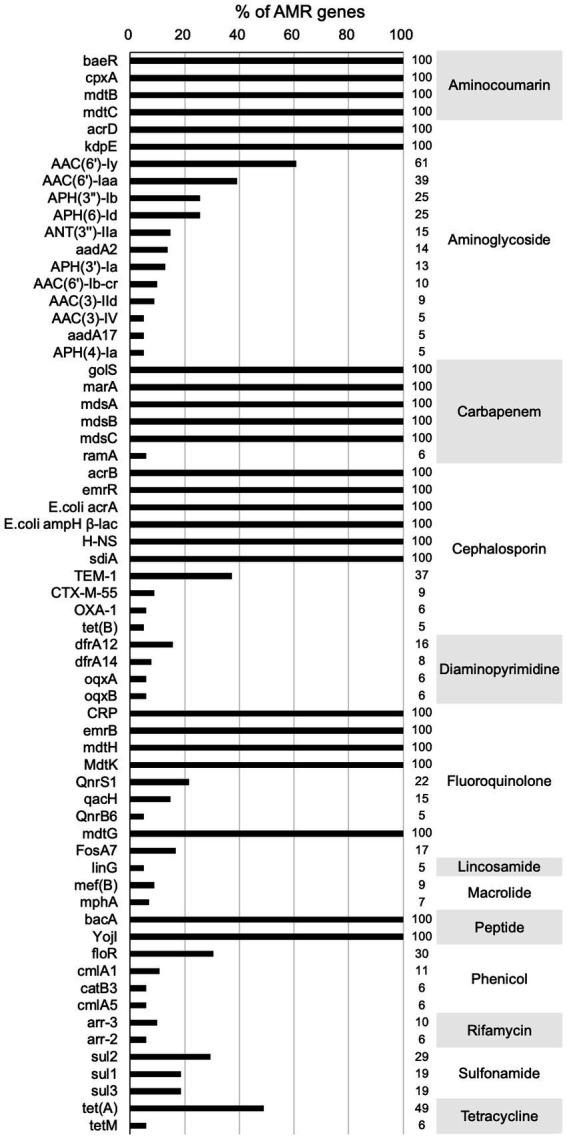
Main antibiotic resistance genes profiles. (The proportion of drug resistance genes ≥5%).

Figures 3, 4 present the findings of antimicrobial resistance gene prediction, revealing a total of 24 drug-resistant genes in all *Salmonella* strains studied: *acrB*, *acrD*, *bacA*, *baeR*, *cpxA*, *CRP*, *emrB*, *emrR*, *E. coli acrA*, *E. coli ampH beta-lactamase*, *golS*, *H-NS*, *kdpE*, *marA*, *mdsA*, *mdsB*, *mdsC*, *mdtB*, *mdtC*, *mdtG*, *mdtH*, *MdtK*, *sdiA*, and *YojI*. Furthermore, 60.8% (62/102) of isolates harbored the *AAC (6′)-Iy gene*, which was responsible for aminoglycoside resistance. Approximately 49.0% (50/102) of strains had the *tet (A)* gene for tetracycline resistance, whereas 39.2% (40/102) carried the *AAC (6′)-Iaa* gene. The *TEM-1* gene was found in 37.3% (38/102) of isolates, the *floR* gene in 30.4% (31/102), and *sul2* gene in 29.4% (30/102). These genes confer resistance to ampicillin/penicillin, florfenicol, and sulphonamides, respectively.

Concerning the distribution of antimicrobial resistance genes among the predicted serotypes based on sequence type, our data revealed that the presence of antimicrobial resistance genes was more prevalent in *S.* Agona, *S.* Derby, *S.* Kentucky, and *S.* Muenster than in the other serotypes, whereas *S.* Aberdeen, *S.* Bareilly, *S.* Hvittingfoss, *S.* Newport, and *S.* Thompson contained the least number of resistance genes (Figure 3, Supplementary Data Sheet 1_“CARD” sheet). Moreover, the distribution of antimicrobial resistance genes in *Salmonella* strains isolated from North Guizhou did not significantly differ from those isolated from South Guizhou (Supplementary Figure S2, *p* > 0.05).

### 3.4 Virulence gene prediction

Supplementary Data Sheet 1 (“VFDB” sheet) provides predictions for virulence genes using the Virulence Factors Database; 35 potential virulence genes were identified. Among the individual *Salmonella* strains, the highest and lowest counts of virulence genes were found in *S. typhimurium* (28 genes) and *S.* Derby (20 genes), respectively. Our data indicated that *cdtB*, which encodes typhoid toxins, was found in *S. Indiana* (5 strains), *S.* Muenster (4 strains), and *S.* Goldcoast (2 strains). In contrast, 10 *S. typhimurium* strains and 7 *S. enteritidis* strains carried the *plasmid-encoded fimbriae* (*Pef; VF0104*) and *Salmonella plasmid virulence* (*Spv; VF0107*) genes, which encode fimbriae and significantly contribute to the virulence of non-typhoidal *Salmonella* strains. Every strain examined possessed key virulence factors from *Salmonella* Pathogenicity Islands 1 and 2 (*SPI-1* and *SPI-2*) and peritrichous flagella (*AI145*), as well as *AcrAB* (*VF0568*), *Agf* (*VF0103*), *Bcf* (*AI058*), *OmpA* (*VF0236*), *Enterobactin* (*IA019*), *AGF* (*AI094*), *Capsule* (*VF0560*), *Enterobactin* (*VF0228*), *MgtBC* (*VF0106*), *RcsAB* (*VF0571*), *SinH* (*VF0400*), *Type 1 fimbriae* (*VF0102*), and *MisL* (*VF0397*).

### 3.5 Plasmid profiles

**Figure 5:**
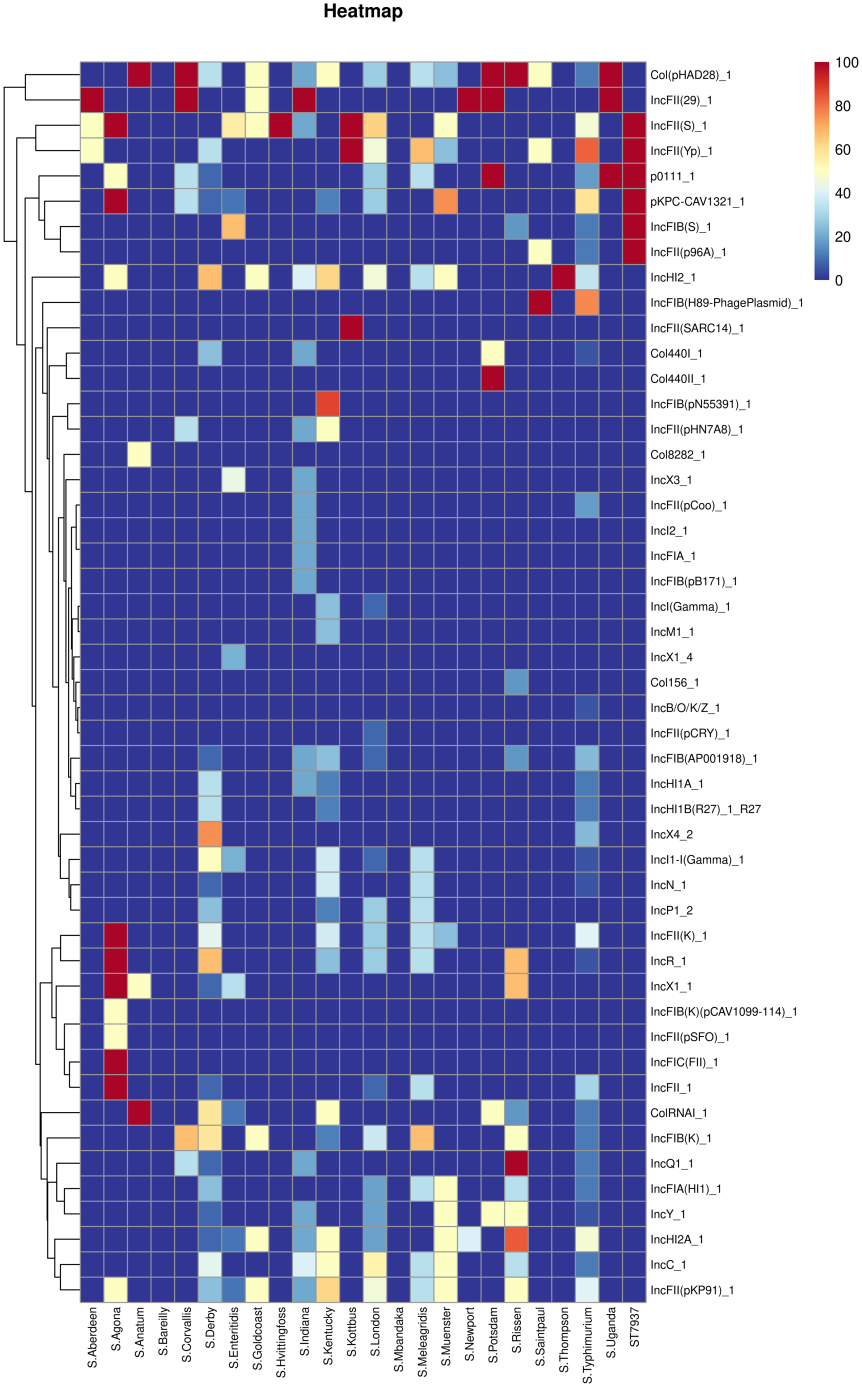
The heatmap of plasmids distribution in different *Salmonella* isolates. Scale 0–100, representing 0–100% proportions.

Figure 5 shows the predicted plasmid types of the studied *Salmonella* isolates. Forty-nine different plasmids were identified in the *Salmonella* isolates. Our results showed that *IncHI2_1* and *Col (pHAD28)_1* were the most prevalent plasmids (present in 32 isolates), followed by *IncFII (pKP91)_1*, *IncFII (S)_1*, and *IncFII (Yp)_1* (*n* = 30 isolates). Additionally, *S.* Agona possessed an average of 10 plasmid types, followed by *S*. Derby (average plasmid types = 8), *S*. Kentucky, *S*. Rissen, and *S. typhimurium* (average plasmid types = 7). In contrast, *S*. Bareilly and *S*. Mbandaka did not harbor any plasmids.

### 3.6 Comparative and correlation analyses

Table 3 shows the Comparative analysis of drug susceptibility, drug resistance genes, virulence genes, and plasmids of 2 3-year periods. We found that the number of drug-resistant agents (3.13 ± 3.10 vs. 5.15 ± 3.10, *p* = 0.002, Table 3) and drug-resistant genes (29.19 ± 5.89 vs. 32.19 ± 6.61, *p* = 0.006, Table 3) of *Salmonella* in 2016–2018 was significantly lower than that in 2019–2021, suggesting a marked escalation in drug resistance over the recent 3 years. On the contrary, the virulence genes of *Salmonella* in 2016–2018 were significantly higher than those in 2019–2021 (23.96 ± 2.41 vs. 22.96 ± 2.07, *p* = 0.035, Table 3), indicating that the virulence of *Salmonella* has declined in recent years. In addition, compared with 2016–2018, the number of plasmids carried by *Salmonella* in 2019–2021 increased, but no statistical difference was found (7.06 ± 5.21 vs. 9.31 ± 7.31, *p* > 0.05, Table 3).

**Table 3:** Comparative analysis of drug susceptibility, drug resistance genes, virulence genes, and plasmids between two groups (2016–2018 vs. 2019–2021).

Items	Groups	*N*	Mean ± std.	*p*-value
MIC(R)_SUM	2016–2018	48	3.13 ± 3.10	0.002
	2019–2021	54	5.15 ± 3.10	
CARD_SUM	2016–2018	48	29.19 ± 5.89	0.006
	2019–2021	54	32.19 ± 6.61	
VFDB_SUM	2016–2018	48	23.96 ± 2.41	0.035
	2019–2021	54	22.96 ± 2.07	
Plasmid_SUM	2016–2018	48	7.06 ± 5.21	0.199
	2019–2021	54	9.31 ± 7.31	

Std: standard deviation; MIC (R)_SUM: In the drug sensitivity test, the total count of antibiotic resistances exhibited by each isolated Salmonella bacterium; CARD_SUM: Utilizing the Comprehensive Antibiotic Resistance Database (CARD), the total counts of antibiotic resistance genes present in each isolated Salmonella bacterium were calculated; VFDB_SUM: Based on the Virulence Factor Database (VFDB), the total number of virulence genes for each Salmonella isolate were calculated; Plasmid_SUM: Employing the PlasmidFinder, the total count of plasmids for every individual Salmonella isolate were calculated.

Supplementary Figure S3 demonstrates the correlation between phenotypic and genotypic antimicrobial resistance. Significant correlations were observed between *Colistin* and *aadA5* (ρ = 0.581, *p* < 0.001), *dfrA17* (ρ = 0.581, *p* < 0.001), *CMY-2*(ρ = 0.286, *p* = 0.004), *FosA3* (ρ = 0.255, *p* = 0.010), and *BalTEM-1* (ρ = 0.255, *p* = 0.010). Supplementary Figure S4 shows the correlation between phenotypic antimicrobial resistance and plasmid. Significant correlations were observed between *Colistin* and *IncFIB (S)_1* (ρ = 0.523, *p* < 0.001), *IncX3_1* (ρ = 0.507, *p* < 0.001), *IncX1_4* (ρ = 0.407, *p* < 0.001), *IncFIA_1* (ρ = 0.286, *p* = 0.004), *IncFIB (pB171)_1* (ρ = 0.286, *p* = 0.004), and *IncI2_1* (ρ = 0.286, *p* = 0.004). Significant correlations were also observed between phenotypic antimicrobial resistance and virulence genes (Supplementary Figure S5), between genotypic antimicrobial resistance and plasmids (Supplementary Figure S6), and between genotypic antimicrobial resistance and virulence genes (Supplementary Figure S7).

### 3.7 Phylogenetic analyses

We performed phylogenetic analyses using MLST and cgMLST.

**Figure 6:**
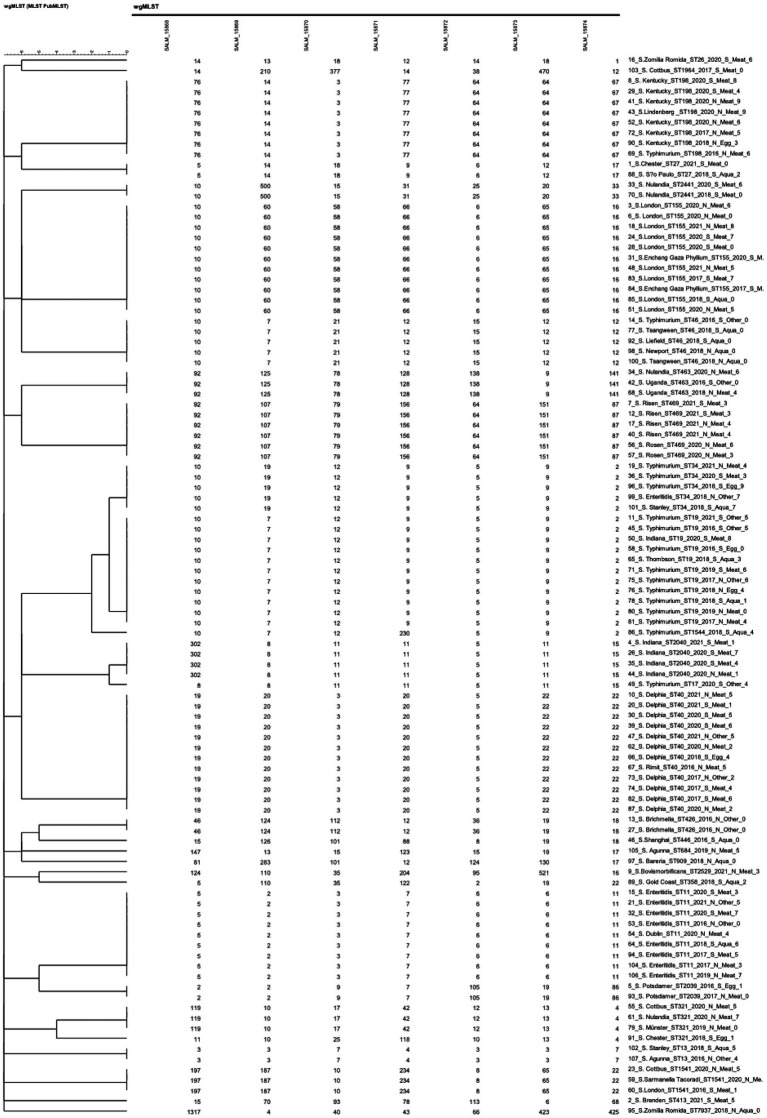
MLST of 102 *Salmonella* isolates.

Figure 6 showed that the 102 *Salmonella* isolates were divided into 28 STs using *in silico* MLST, which revealed that ST40 was the most common (*S.* Derby, *n* = 12), followed by ST19 (*S. typhimurium*, *n* = 11), ST155 (*S.* London, *n* = 11), ST11 (*S. enteritidis*, *n* = 9), ST198 (*S.* Kentucky, *n* = 8), ST469 (*S.* Rissen, *n* = 6), ST34 (*S. typhimurium*, *n* = 5), ST46 (*S.* Newport, *n* = 5), ST321 (*S.* Muenster, *n* = 4), and ST2040 (*S. Indiana*, *n* = 4). Among the 28 STs, 14 were observed in 2 or more regions. ST155, ST40, ST19, ST11, and ST34 appeared in more than five regions, indicating that these five STs were prevalent and had the potential for transregional infection.

Statistics of the isolation sources revealed that (1) the 65 isolates from “meat and meat products” comprised 19 different STs, among which ST155 had the highest number of 10 isolates, followed by ST40 (*n* = 9) and ST198 (*n* = 7); (2) the 20 isolates from “aquatic products” belonged to 13 STs, among which ST46 had the highest number (*n* = 5), followed by ST11 (*n* = 2), ST19 (*n* = 2), and ST426 (*n* = 2); (3) the 7 isolates from “eggs and egg products” contained 6 STs, among which ST19 had the highest number (*n* = 2); and (4) the 10 isolates from “other” belonged to 7 different STs. Here, we defined “the STs variation index” as the number of STs/isolates. Thus, the ST variation indexes of “meat and meat products,” “aquatic products,” “eggs and egg products,” and “other” were 29.2, 65.0, 85.7, and 70%, respectively.

The statistics of isolation years showed that the major ST differed every year between 2016 and 2021. ST19 (*n* = 7), ST40 (*n* = 5), and ST46 (*n* = 5) were the major STs for 2016, 2017, and 2018, respectively (*n* = 48), while ST155 (*n* = 8), ST40 (*n* = 7), and ST469 (*n* = 6) were the major ST for 2019, 2020, and 2021, respectively (*n* = 54).

Comparisons of MLST and serotyping results showed that, among the top 10 MLST types mentioned above, only ST469 and ST2040 comprised one serotype. Approximately one-third (32/102) of the serotypes did not match their STs. Results indicated that MLST exhibited higher accuracy compared with serotyping. Moreover, two serotypes included more than one ST: 17 isolates with serotype Typhimurium contained three STs (ST19, ST34, and ST1544), and five isolates of *S. Indiana* contained two STs (ST17 and ST2040).

We performed cgMLST analysis on the 102 isolates based on their genomic sequences. Allelic differences were calculated with assembly-free and assembly-based allele calling using default settings. Supplementary Figure S8 illustrates the relatedness between all sequenced strains via CL, revealing that the 102 *Salmonella* isolates were grouped into 96 clusters. Supplementary Data Sheet 2 shows that 3,002 target loci (Garcia-Soto et al., 2021) were identified in the *Salmonella* genome. We found an allelic difference of 99.2% (2,979/3002) between the 102 isolates.

## 4 Discussion

*Salmonella* infection is a major public health concern in Guizhou Province. In this study, we first described the antimicrobial and phylogenetic characteristics of foodborne Salmonella in Guizhou Province between 2016 and 2021.

### 4.1 Distribution

In our study, the proportion of *Salmonella* was higher in “meat and meat products” (63.70%; 65/102) than in “aquatic products” (19.60%; 20/102) and “other origin” samples (9.80%; 10/102). Meat and meat products have been identified as the primary carriers of *Salmonella*, which are pathogenic to humans (Liu et al., 2021). Generally, the prevalence of *Salmonella* in samples collected from meat and meat products is 12–19% in China (Li et al., 2013; Yang et al., 2019; Jiang et al., 2021; Liu et al., 2021). The contamination of meat products with *Salmonella* can occur at various stages within the retail food chain—from breeding on farms to slaughter, transportation, and eventually sale in different locations and areas. It is crucial to emphasize the control and epidemiological monitoring of *Salmonella* throughout the retail food system as a priority to ensure consumer safety.

Serotype prediction analysis revealed that *S. typhimurium* was the most prevalent serotype among *Salmonella* isolates. According to Supplementary Figure S1, *S. typhimurium* was detected in all four main sources: meat and meat products, eggs and egg products, aquatic products, and other-origin products. In a previous study conducted in China, *S. typhimurium* has also been identified as the predominant isolate in pork meat (Campos et al., 2019), egg samples (Hu et al., 2021), and aquatic food products (Yang et al., 2015). Additionally, in our study, no single source covered all predicted serotypes. Approximately 70% of predicted serotypes were found in “meat and meat products.” *S.* Rissen (*n* = 6) and *S. Corvallis* (*n* = 3) were found only in meat and meat products. *S.* Newport (*n* = 5) and *S.* Aberdeen (*n* = 2) were only found in aquatic products. These observations suggest that cross-contamination of local retail foods may contribute to the presence of *S. typhimurium*. However, different *Salmonella* serotypes exhibit different source preferences.

Moreover, we found that approximately one-third of serotypes determined through agglutination did not correspond to the serotypes identified via MSLT. Generally, the discriminatory power of MLST is comparable to or better than traditional serotyping. If there is a discrepancy between the slide agglutination test and MLST outcomes, many studies would generally lean toward MLST results owing to their superior resolution and reproducibility (Achtman et al., 2012; Pearce et al., 2018; Uelze et al., 2020; Yan et al., 2021). In our study, over 99% (101/102) had their predicted serotypes derived from sequencing types. Thus, in the same way, we chose to analyze the predicted serotypes. MLST is a more reliable and precise method for identifying bacterial strain relationships because it relies on the sequences of multiple housekeeping genes and can even distinguish closely related strains (Yan et al., 2021). However, MLST detects changes at the DNA level that cannot be inferred from the phenotype, such as serotyping or multi-locus enzyme electrophoresis (Uelze et al., 2020). The decision may also depend on the specific context, nature of the discrepancy, and other available evidence. For example, the slide agglutination test results matched other phenotypic or biochemical characteristics of the isolate. If the MLST results do not, additional tests or analyses may be necessary. Here, we illustrated the advantages of using molecular typing methods and routine surveillance in China.

### 4.2 MDR

Our findings revealed that a significant proportion (66.7%) of the *Salmonella* isolates examined (*n* = 102) displayed resistance to at least three classes of antimicrobial agents, indicating MDR. This high percentage is alarming, as it indicates a continuous increase in the prevalence of MDR *Salmonella* strains in retail foods and their surrounding environments. The increase in the MDR rate could potentially be attributed to the inappropriate and excessive use of antibiotics on animal farms for therapeutic and prophylactic purposes (Liu et al., 2021), which contributes to the development and spread of antimicrobial resistance among *Salmonella* strains, ultimately leading to higher MDR rates. Furthermore, many *Salmonella* isolates demonstrated resistance to ampicillin and tetracycline, which are antimicrobial agents widely used in animal farms worldwide (Lekagul et al., 2019). Resistance to quinolones and beta-lactams, two important antimicrobial classes used to treat salmonellosis, has also been identified in numerous *Salmonella* isolates (Castanheira et al., 2020). This substantially threatens public health as these drugs are currently considered the preferred treatment options. Resistance to colistin, previously regarded as a last-resort therapeutic option for treating carbapenem-resistant Enterobacteriaceae, has been reported in several *Salmonella* isolates.

Moreover, a concerning trend emerged during 2019 and 2021, marked by a substantial increase in the prevalence of drug-resistant agents and genes in *Salmonella*. The rise in resistance not only underscores the evolving nature of *Salmonella* but also highlights the need for enhanced surveillance and innovative strategies in antibiotic management in Guizhou. This trend poses significant implications for both healthcare practices and food safety protocols in the province, calling for a coordinated response to address this emerging public health issue.

In addition, noteworthy differences exist between Guizhou’s southern and northern regions regarding population demographics and economic development. The southern areas have a population of approximately 20.2 million, with a higher concentration of ethnic minorities, such as the Miao, Dong, and Buyi communities. These regions generally display lower levels of economic development. In contrast, the northern region has a population of about 18.4 million and is inhabited by more Han Chinese and fewer ethnic minorities. This area had an approximately 1.7 times higher GDP than the south (150 vs. 89 USD billion in 2020) (Guizhou Provincial Bureau of Statistics, 2021; Guizhou Provincial Bureau of Statistics NBS Survey Office in Guizhou, 2021). Although these disparities between the north and south could potentially influence local *Salmonella* characteristics (Bhattacharya et al., 2023; Wang et al., 2023), our study did not find any significant differences between the two regions.

### 4.3 Antimicrobial resistance genes

The prediction of antimicrobial resistance genes in the studied *Salmonella* isolates revealed the presence of several genes that could contribute to phenotypic resistance. The results of this study showed that all isolates harbored 24 drug-resistant genes: *acrB*, *acrD*, *bacA*, *baeR*, *cpxA*, *CRP*, *emrB*, *emrR*, *E. coli acrA*, *E. coli ampH beta-lactamase*, *golS, H-NS*, *kdpE*, *marA*, *mdsA*, *mdsB*, *mdsC*, *mdtB*, *mdtC*, *mdtG*, *mdtH*, *MdtK*, *sdiA*, and *YojI*. Few studies have reported that all *Salmonella* strains in a certain area in China are resistant to more than 20 drug-resistant genes (Liu et al., 2021; Chen et al., 2022; Sun et al., 2022). Additionally, other notable antimicrobial-resistant genes, such as *tet (A)* (49.0%, 50/102), which encodes resistance to tetracycline, and *AAC (6′)-Iaa* (39.2%, 40/102), which encodes resistance to aminoglycosides, were also identified. However, the presence of these genes in bacterial genomes does not guarantee phenotypic resistance or vice versa (Piddock, 2016). Antimicrobial resistance cannot be attributed solely to the presence or absence of resistance genes. Additional factors, including enzyme activation, target modification/protection, regulation of gene expression related to antimicrobial resistance, and even changes in the cell wall charge, significantly contribute to the development of antimicrobial resistance. Consequently, when comparing only the presence of antimicrobial-resistant genes, some degree of discordance is anticipated. Given the multitude of variables and intricate connections between genotypic and phenotypic data, a comprehensive evaluation of genotype–phenotype correlations provides a more accurate and comprehensive understanding of antimicrobial resistance (Liu et al., 2020). Therefore, phenotypic analysis was necessary to confirm the antimicrobial-resistant profiles of the studied isolates.

### 4.4 Virulence genes

In our study, several detected plasmids carried virulence genes associated with the virulence system of *Salmonella*. Notably, *cdtB*, which encodes typhoid toxins, was detected in 5 *S. Indiana*, 4 *S.* Muenster, and 2 *S.* Goldcoast strains. Moreover, the *spv* and *pef* genes were identified in 10 *S. typhimurium* and 7 *S. enteritidis* isolates. The detection of the *cdtB* gene in non-typhoidal *Salmonella* strains is notable because its presence has been linked to severe instances of bloodstream infections and invasive conditions in humans (Xu et al., 2020). Moreover, the spv locus has been closely linked to strains that cause non-typhoidal bacteremia (Fierer, 2022). *Pef* also plays an essential role in the virulence mechanisms of non-typhoidal *Salmonella* strains (Ben Hassena et al., 2021). The presence of *Salmonella* strains carrying these virulence factors in the retail food supply chain poses a considerable threat to public health, potentially leading to severe illness in consumers.

Meanwhile, an intriguing pattern emerged in the behavior of *Salmonella* strains over two distinct timeframes: 2016–2018 and 2019–2021. During the earlier period, the virulence genes of *Salmonella* were significantly higher, suggesting a greater potential for causing severe illness. However, this trend was reversed in the latter period, where there was a notable decrease in virulence genes. In contrast, this period saw a significant rise in the number of drug-resistant agents and drug-resistant genes, signaling an emerging challenge in combating these infections with standard antibiotics. This shift, with decreasing virulence but increasing drug resistance, highlights the complex and evolving nature of *Salmonella* in the region, necessitating a multifaceted and dynamic approach to public health interventions and antibiotic management strategies in Guizhou.

### 4.5 Plasmid

Analysis of plasmid distribution among *Salmonella* isolates revealed the presence of 49 different plasmids. The most prevalent plasmids detected were *IncHI2_1* and *Col (pHAD28)_1*, which were observed in 32 isolates, followed by *IncFII (pKP91)_1*, *IncFII (S)_1*, and *IncFII (Yp)_1*, which were present in 30 isolates. Notably, transferable plasmids, such as *IncQ1_1*, *IncR_1*, *IncHI2_1*, and *IncX1_1*, were detected in the MDR strains. These mobile plasmids confer resistance against various antimicrobial groups, encompassing β-lactams, aminoglycosides, sulfonamides, tetracycline, and others (Chen et al., 2016; McMillan et al., 2020). Moreover, we detected the *IncFIB (S)_1* and *IncFII (S)_1* plasmids, which encode virulence factors, in the four *S. enteritidis* samples. These plasmids have been documented in *S. enteritidis* isolates sourced from clinical and food samples (Mansour et al., 2020; Liu et al., 2021).

### 4.6 Correlation analyses

Our study observed that the colistin significantly correlated to *aadA5* and *drfA17* (ρ > 0.5, Supplementary Figure S3). Currently, the *aadA5* and *drfA17* genes are not recognized as causative agents for colistin resistance. These genes are predominantly linked to resistance against other antibiotic types: The *aadA5* gene is known for conferring resistance to aminoglycosides (Wang et al., 2017). Similarly, the *drfA17* gene is primarily associated with resistance to trimethoprim (Gu et al., 2020). Like *aadA5*, *drfA17* does not have a known role in colistin resistance. Colistin resistance, in contrast, is frequently connected to alterations in the bacterial cell membrane, especially changes in the lipid A structure of the lipopolysaccharide layer in Gram-negative bacteria. Genes commonly implicated in colistin resistance include *mcr-1* to *mcr-10* and those involved in two-component regulatory systems, such as PmrAB and PhoPQ (Li et al., 2022). The significant correlation between colistin resistance and the presence of *aadA5* and *drfA17* genes, evidenced by a correlation coefficient (ρ) exceeding 0.5, is noteworthy given their traditional lack of association with colistin resistance. Potential explanations for this correlation could include co-selection, cross-resistance, genetic linkage, and novel resistance mechanisms (Pal et al., 2017). Further investigation and experimental validation are needed to comprehend the underlying mechanisms of these correlations.

### 4.7 Phylogenetic analyses

In this study, we compared the evolutionary trees generated by MLST and cgMLST. Both methods yielded similar clustering results. However, cgMLST provides a higher resolution, allowing the detection of minor differences between isolates and generation of more detailed clustering patterns. MLST, which relies on the analysis of 7 housekeeping genes, is a precise and dependable typing method suitable for routine microbial surveillance (Yan et al., 2021). Since MLST is advantageous for establishing associations with specific serotypes, high-resolution molecular methods, including WGS-based cgMLST, cannot easily replace this method (Kimura, 2018). Different cgMLST classification schemes influence *Salmonella* strains (Li et al., 2008). Among the various cgMLST schemes available, the widely accepted 3,002 core loci cgMLST scheme for *Salmonella*, promoted in EnteroBase, is favored owing to its default settings that align with the preferences of many microbiologists, promoting consistency and accuracy between laboratories and jurisdictions. With over 270,000 *Salmonella* genomic sequences included, EnteroBase facilitates the cgMLST analysis of *Salmonella* and provides robust data for pathogen evolution analysis (Achtman et al., 2021). Based on thousands of genomic alleles, WGS-based cgMLST has shown significant potential for enhancing typing accuracy and facilitating convenient data sharing and comparison across international laboratories (Yan et al., 2021). The advancement of WGS-based cgMLST for traceability typing holds immense promise for improving our understanding and management of microorganisms with significant public health and ecological implications.

## 5 Conclusion

This study presents an in-depth analysis of *Salmonella* in retail food products from 2016 to 2021 in Guizhou province, China, uncovering a marked increasing trend in MDR *Salmonella* strains. Meanwhile, the resistance rates differed among strains and food sources, with strains from meat products showing significantly higher drug resistance than those from other sources. Our data also showed that *S. typhimurium* and *S. enteritidis* were the most prevalent strains, with a notable presence of MDR strains and key virulence genes such as *cdtB*, *Pef*, and *Spv*. This study underscores the alarming escalation in both the number of drug-resistant agents and resistance genes in *Salmonella*, particularly in meat-derived strains, highlighting the urgent need for enhanced surveillance measures and innovative antibiotic management strategies in Guizhou, China.

## Data availability statement

The original contributions presented in the study are included in the article/Supplementary material, further inquiries can be directed to the corresponding authors. The data presented in the study are deposited in the NCBI’s Sequence Read Archive repository, accession number PRJNA1063165 (SAMN39337012-SAMN39337113).

## Ethics statement

This study only involved food samples. No ethical approval was deemed required for the experiments conducted in current study.

## Author contributions

LZ: Funding acquisition, Resources, Writing – review & editing, Conceptualization, Investigation, Methodology, Project administration, Supervision, Validation. QY: Data curation, Formal analysis, Writing – review & editing, Visualization. QZ: Writing – review & editing. JW: Writing – original draft. GL: Writing – original draft. JX: Writing – original draft. JH: Writing – original draft. YZ: Writing – original draft. TZ: Writing – original draft. HZ: Data curation, Formal analysis, Funding acquisition, Resources, Software, Writing – original draft, Writing – review & editing. SL: Writing – original draft.

## Glossary

MDR: Multi-drug resistant
MALDI-TOF-MS: Matrix-assisted laser desorption ionization time-of-flight mass spectrometry
AMP: Ampicillin
AMS: Ampicillin/sulbactam
TET: Tetracycline
CFZ: Cefazolin
CTX: Cefotaxime
CFX: Cephalosporin cefoxitin
CAZ: Ceftazidime
IMI: Imipenem
GEN: Gentamicin
CT: Colistin (Polymyxin E)
AZM: Azithromycin
NAL: Nalidixic acid
CIP: Ciprofloxacin
CHL: Chloramphenicol
SXT: Trimethoprim/sulfamethoxazole
CLSI: Clinical Laboratory Standards Institute
CARD: Comprehensive Antibiotic Resistance Database
VFDB: Virulence Factor Database
TraNet: National Foodborne Disease Molecular Tracing Network
MLST: Multi-locus sequence typing
cgMLST: Core genome MLST
