# Supplementary material for: Antimicrobial resistance and genomic investigation of Salmonella isolated from retail foods in Guizhou, China
Source: Front Microbiol. 2024 Mar 6;15:1345045. doi: 10.3389/fmicb.2024.1345045 (PMC10951074; doi:10.3389/fmicb.2024.1345045)
Supplement: Supplementary file 1 [file Data_Sheet_1.ZIP › Supplementary file_Fig S1-Fig S8.docx]

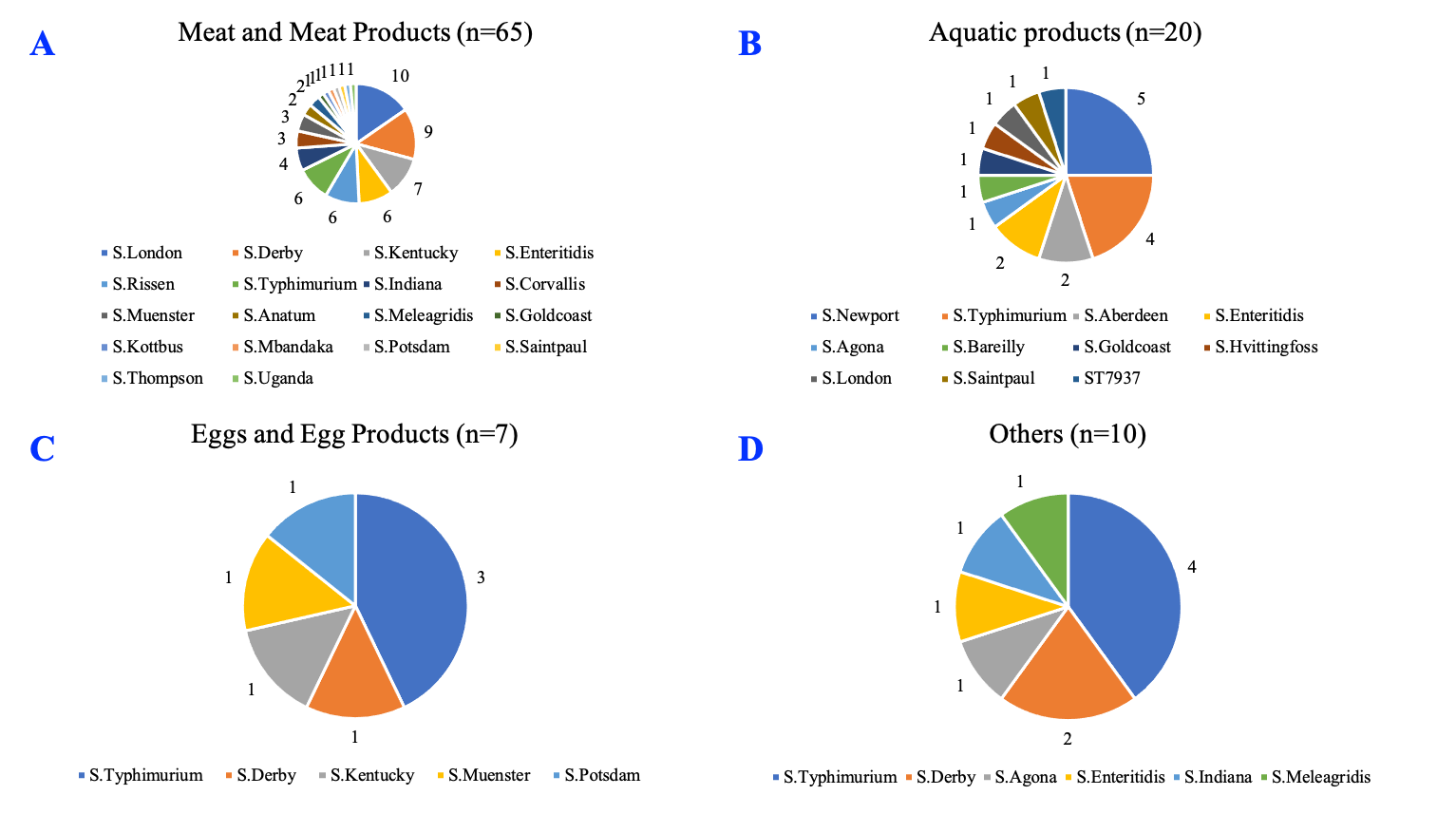


**Figure S1** Distribution of different types of *Salmonella* in 4 types of food.


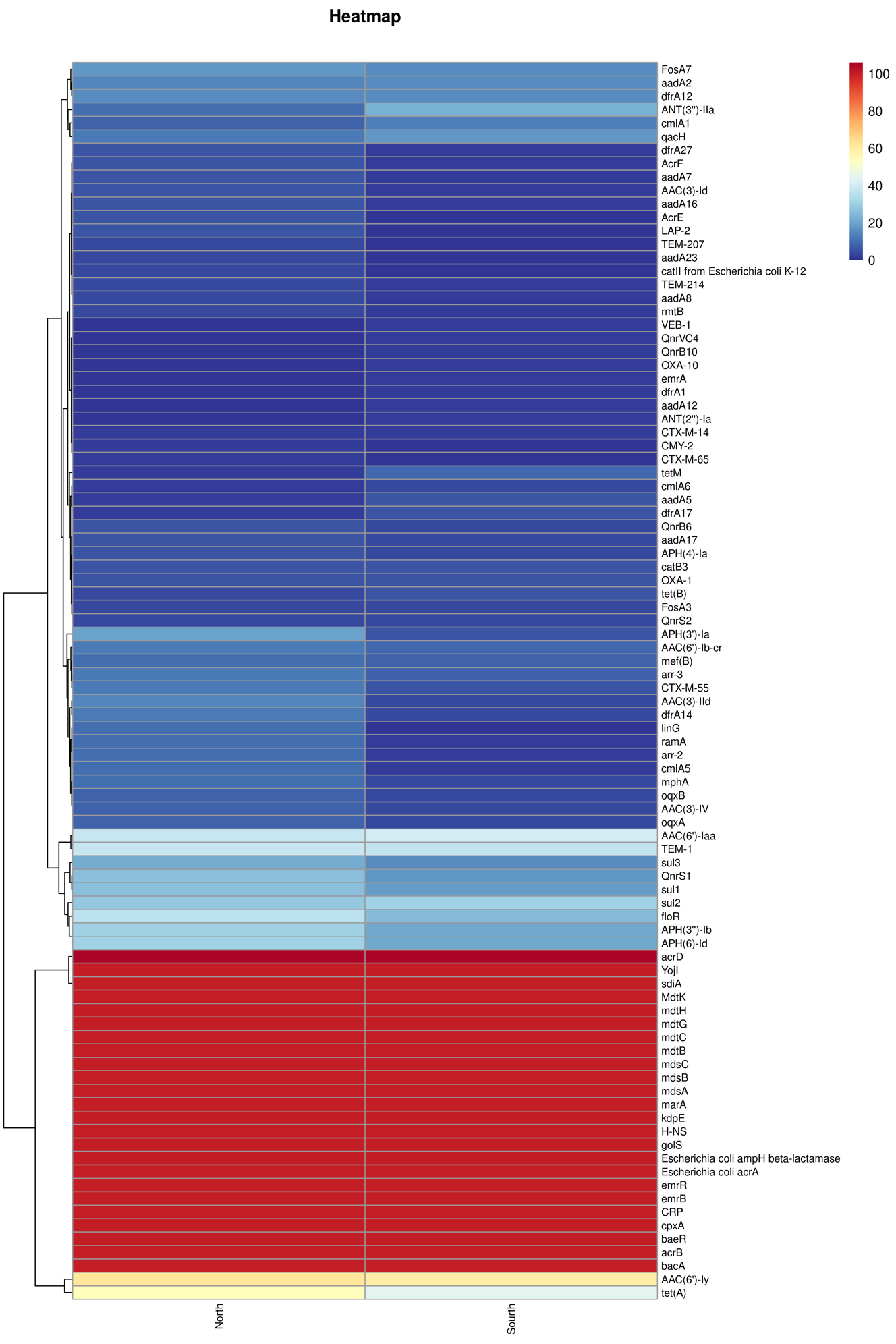


**Figure S2.** The heatmap of antimicrobial resistance genes in the studied Salmonella strains according to sampling sites. Scale 0-100, representing 0%-100% proportions.

**
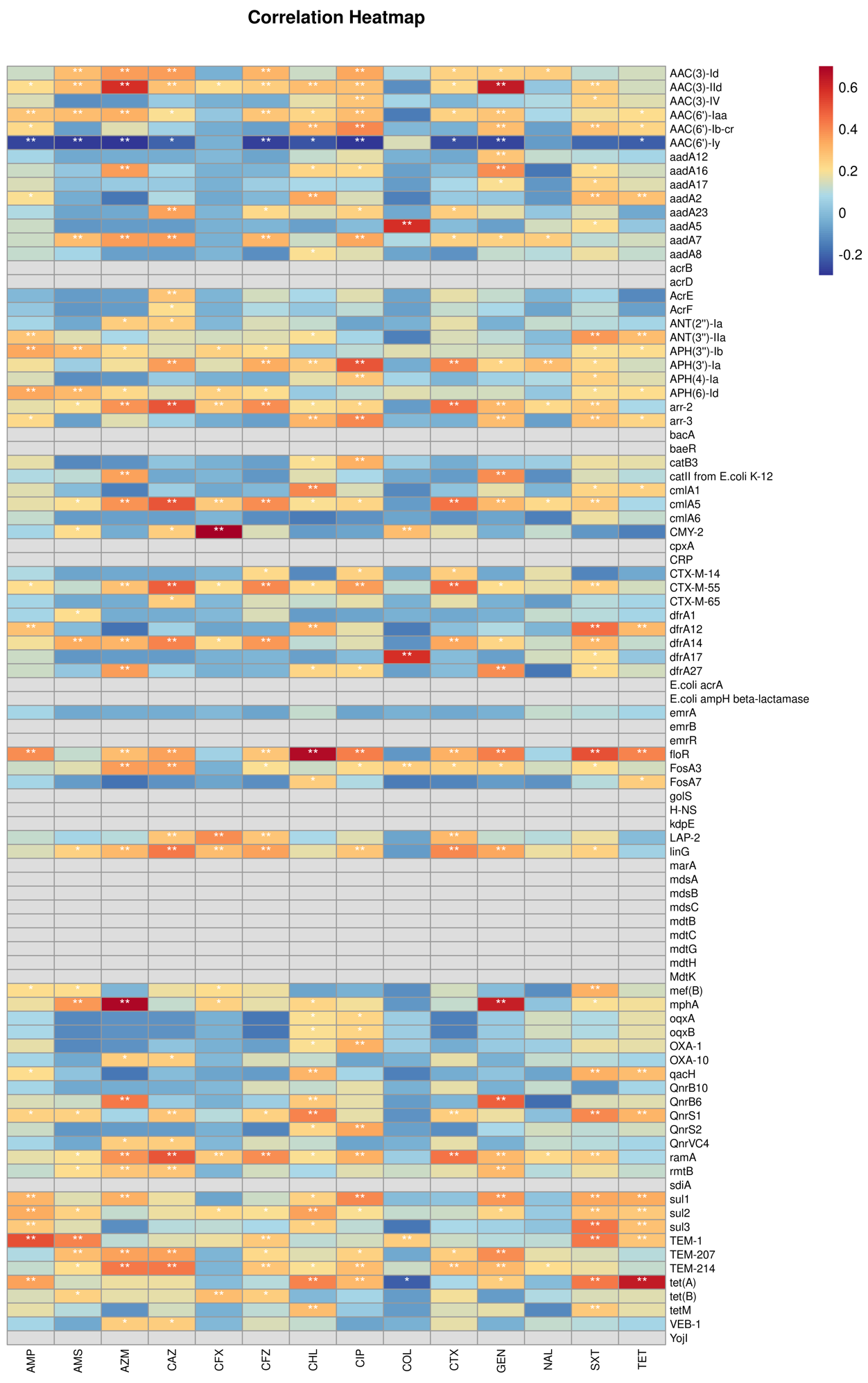
**

**Figure S3.** Correlation between phenotypic antimicrobial resistance and genotypic antimicrobial resistance. Scale 0-1, representing correlation coefficient (ρ) ranging from 0-1.

**
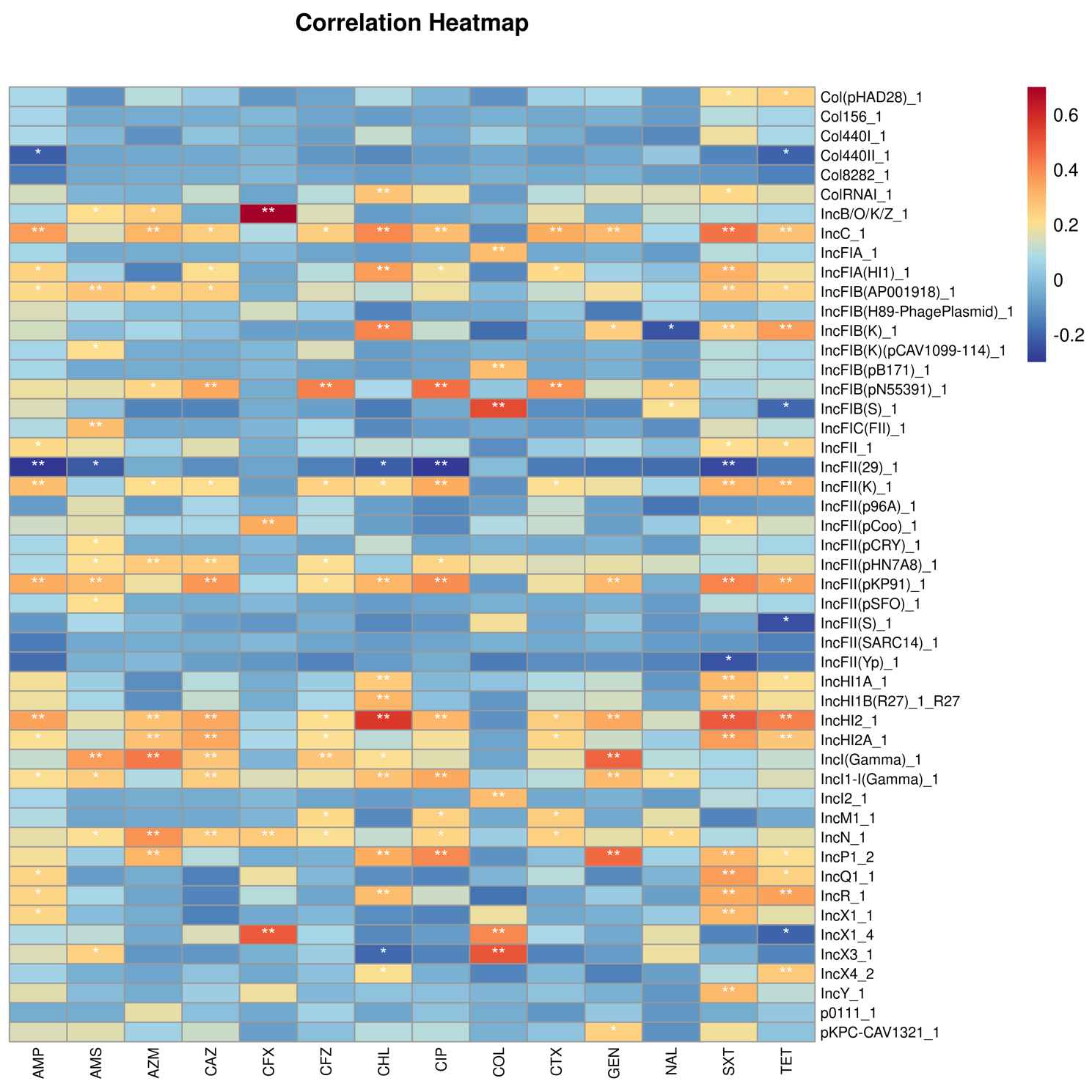
**

**Figure S4.** Correlation between phenotypic antimicrobial resistance and plasmid. Scale 0-1, representing correlation coefficient (ρ) ranging from 0-1.

**
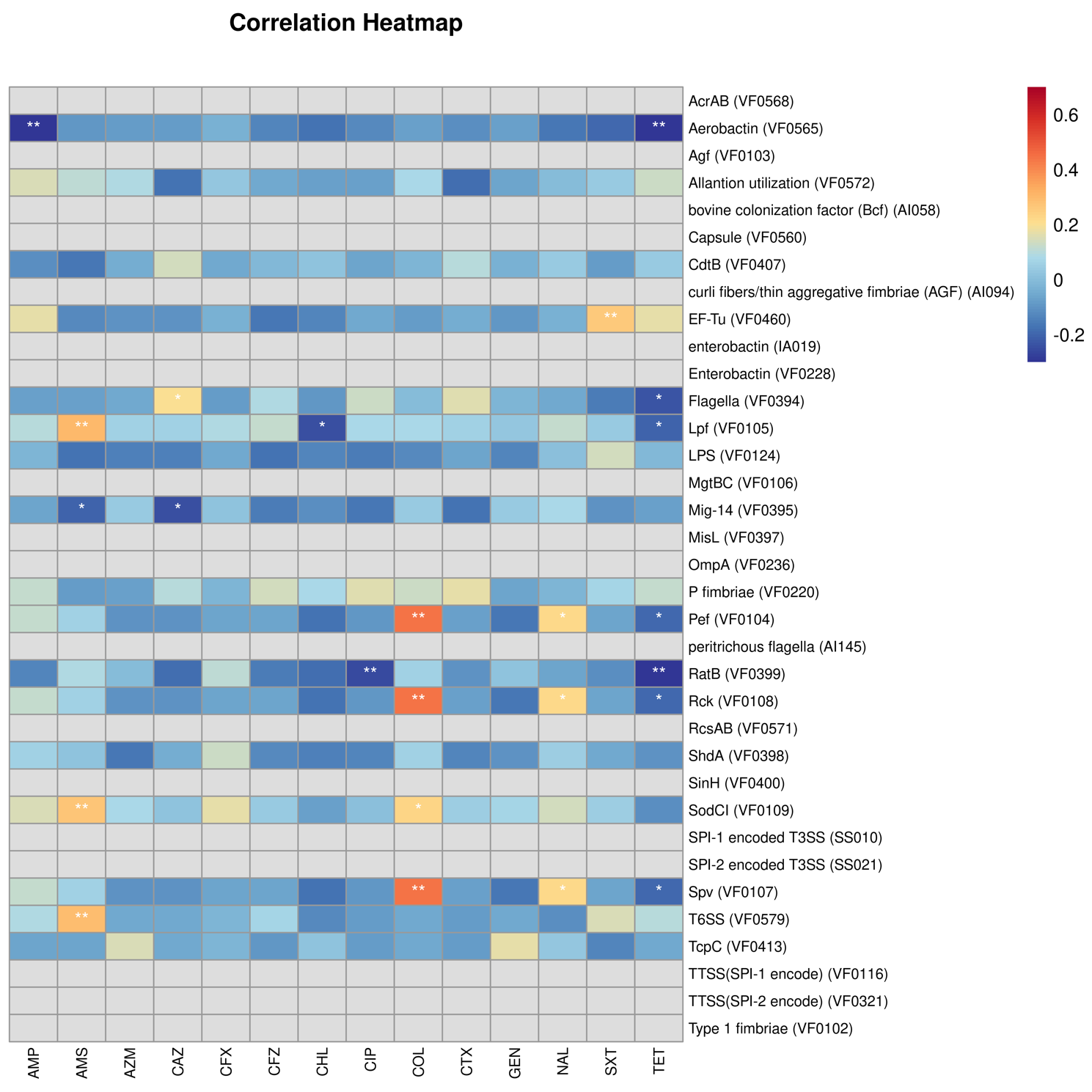
**

**Figure S5.** Correlation between phenotypic antimicrobial resistance and virulence genes. Scale 0-1, representing correlation coefficient (ρ) ranging from 0-1.

**
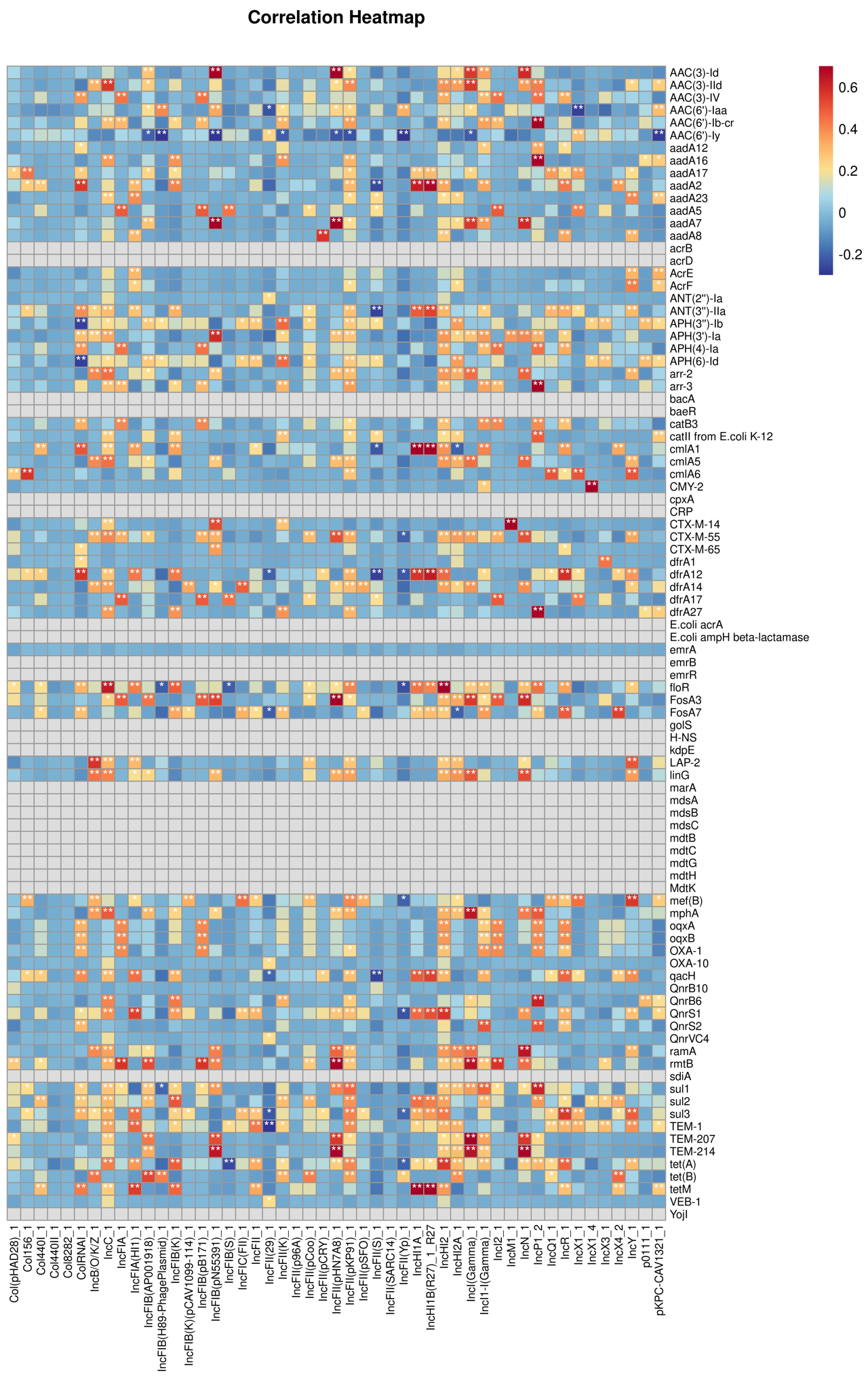
**

**Figure S6.** Correlation between genotypic antimicrobial resistance and plasmid. Scale 0-1, representing correlation coefficient (ρ) ranging from 0-1.

**
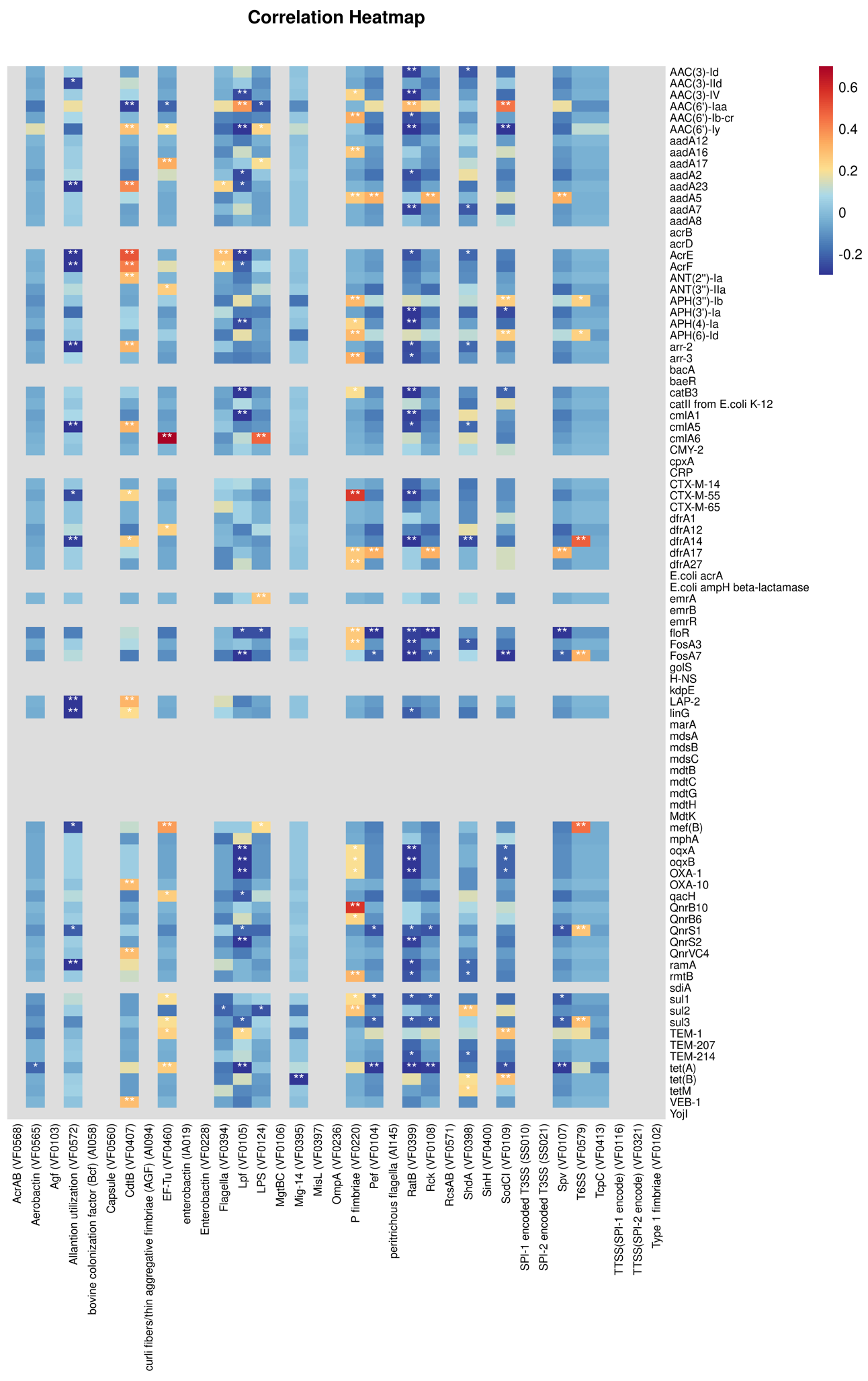
**

**Figure S7.** Correlation between genotypic antimicrobial resistance and virulence genes. Scale 0-1, representing correlation coefficient (ρ) ranging from 0-1.

**
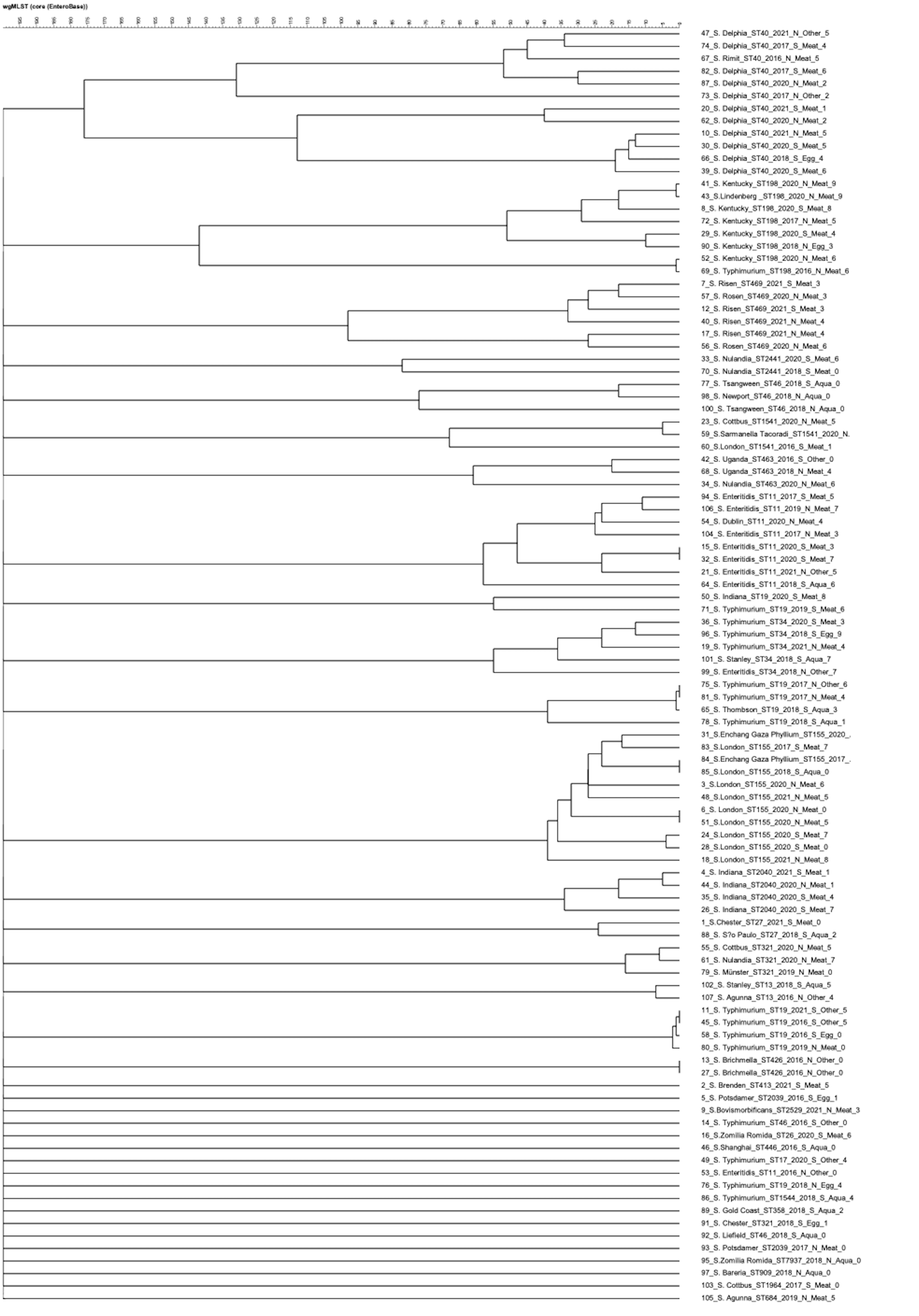
**

**Figure S8** cgMLST of 102 *Salmonella* isolates.
