# Supplementary figures and images for: Antimicrobial resistance and genomic investigation of Salmonella isolated from retail foods in Guizhou, China
Source: Front Microbiol. 2024 Mar 6;15:1345045. doi: 10.3389/fmicb.2024.1345045 (PMC10951074; doi:10.3389/fmicb.2024.1345045)

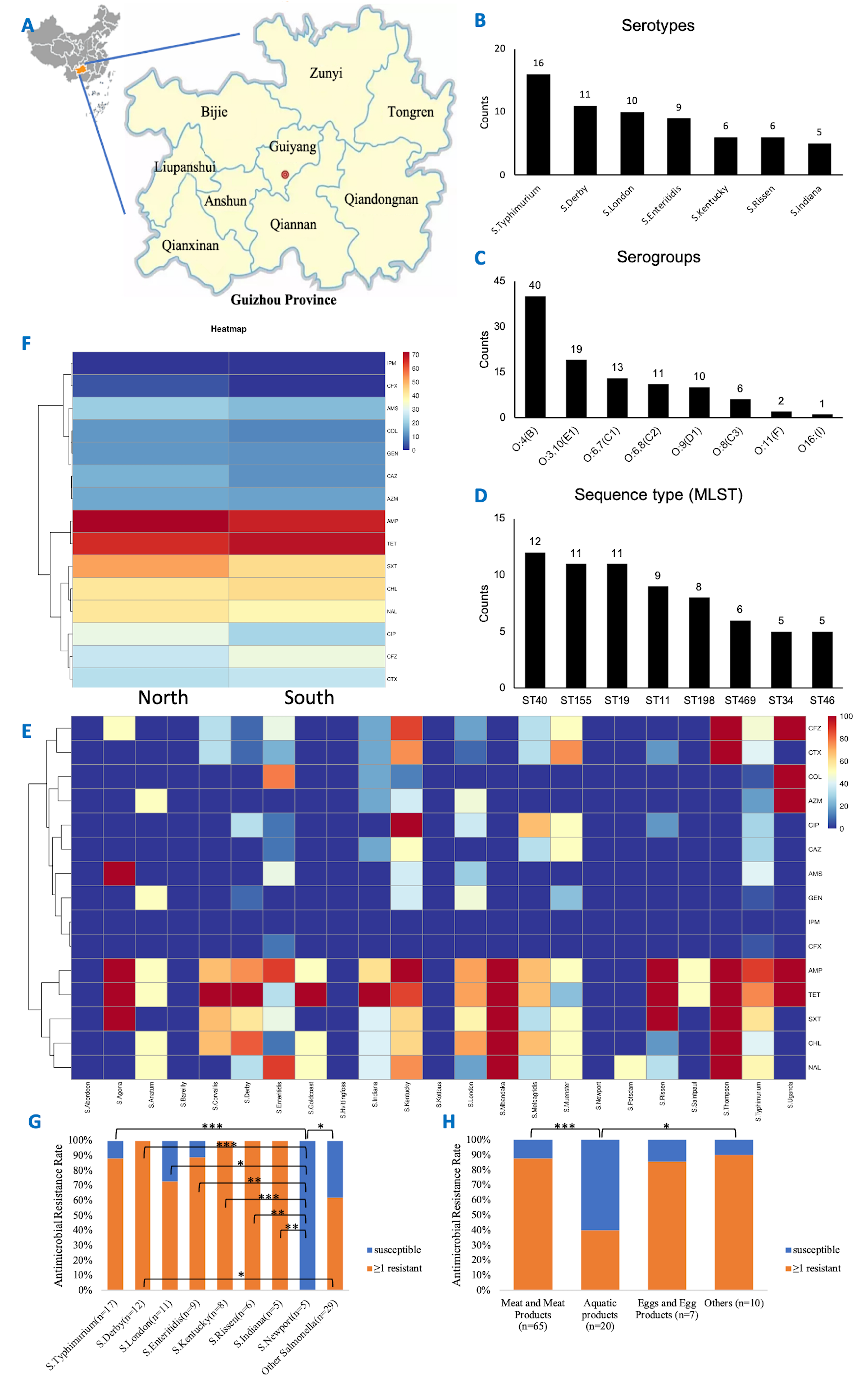

Supplement: Supplementary file 1 [file Data_Sheet_1.ZIP › Fig1_classification_AR patterns_A-H***.tif]

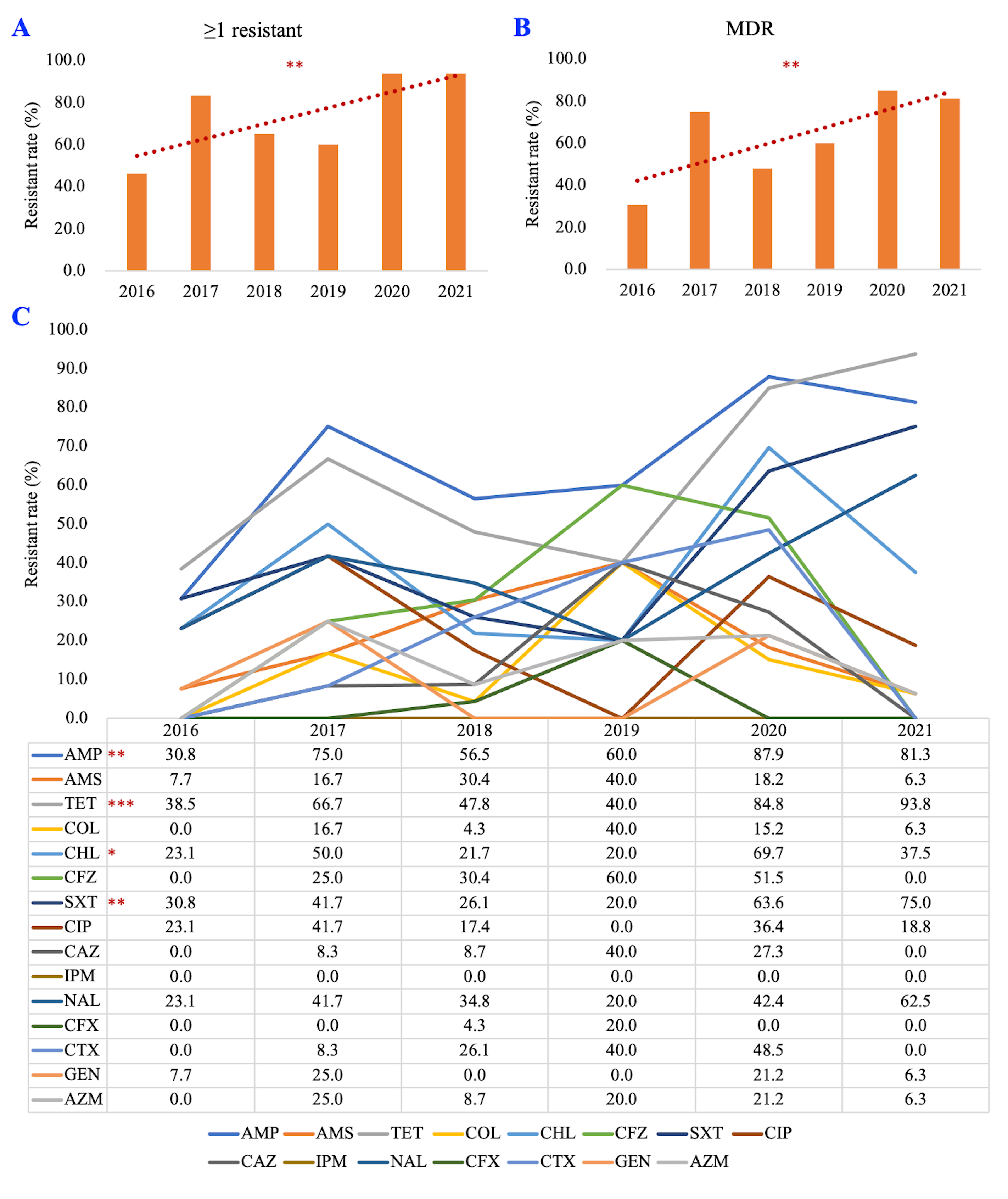

Supplement: Supplementary file 1 [file Data_Sheet_1.ZIP › Fig2_Stratified analysis by year for AST.tif]

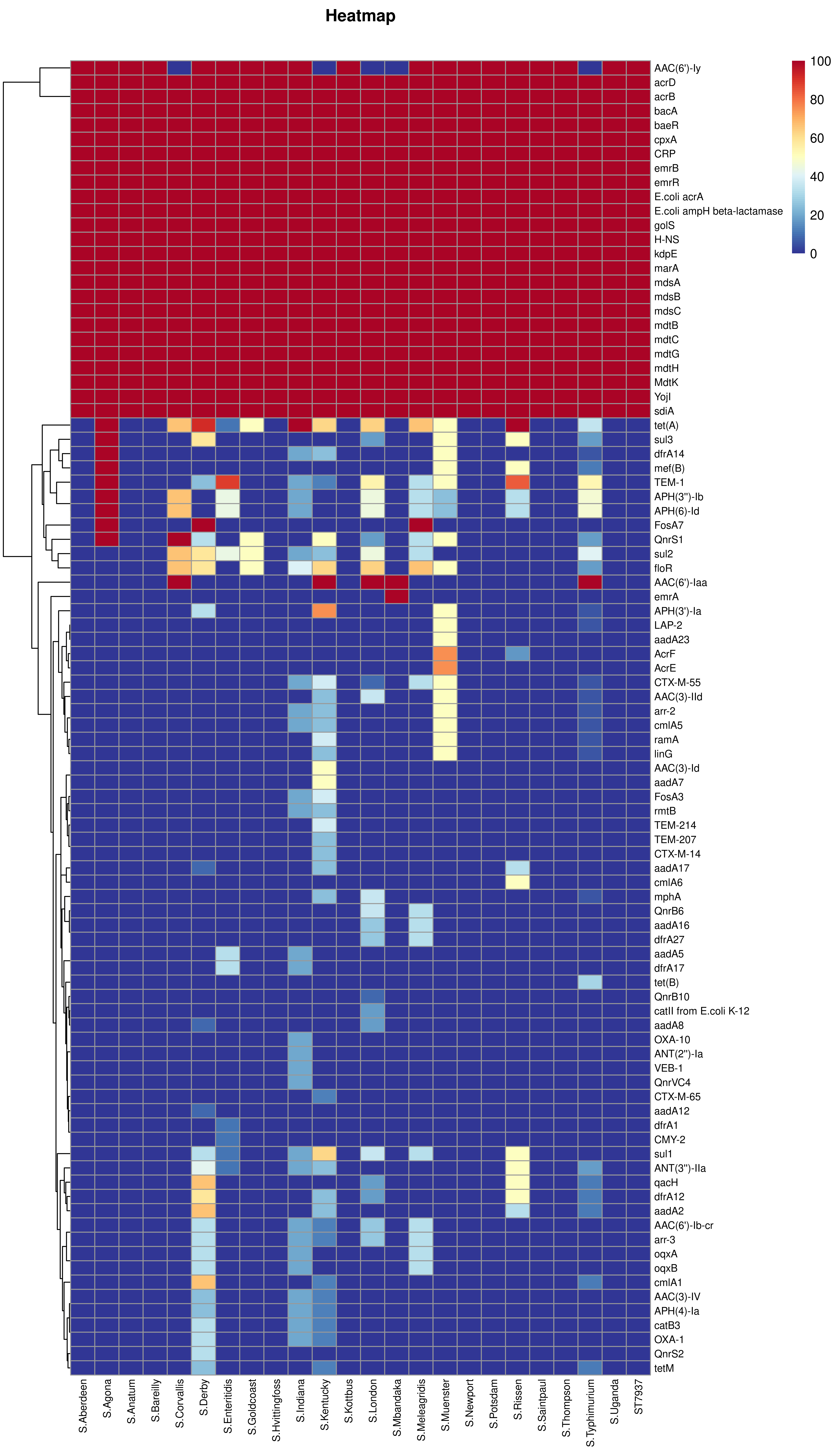

Supplement: Supplementary file 1 [file Data_Sheet_1.ZIP › Fig3_CARD_different Slamonella strains.tif]

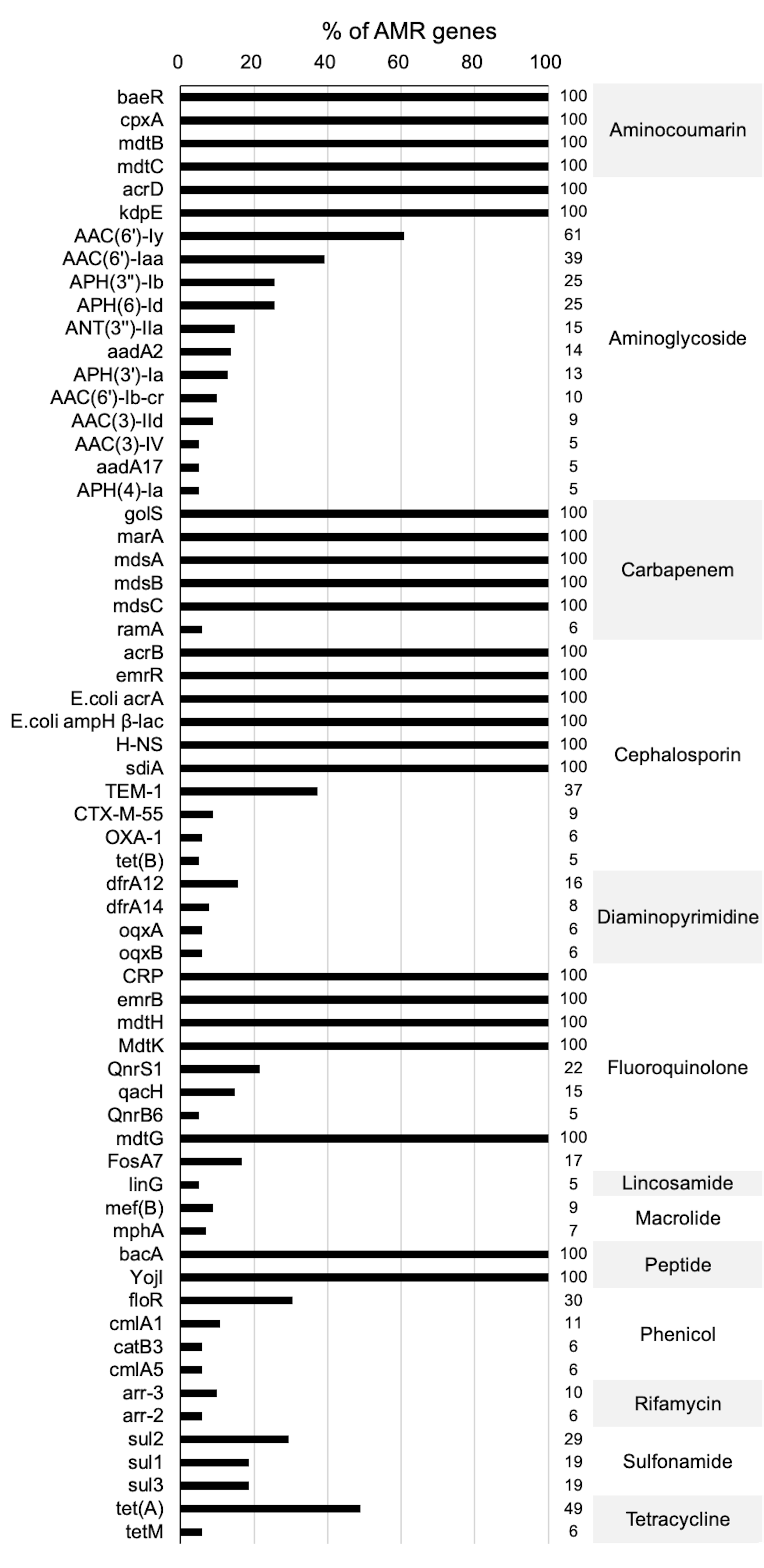

Supplement: Supplementary file 1 [file Data_Sheet_1.ZIP › Fig4_main CARD_ profiles.tif]

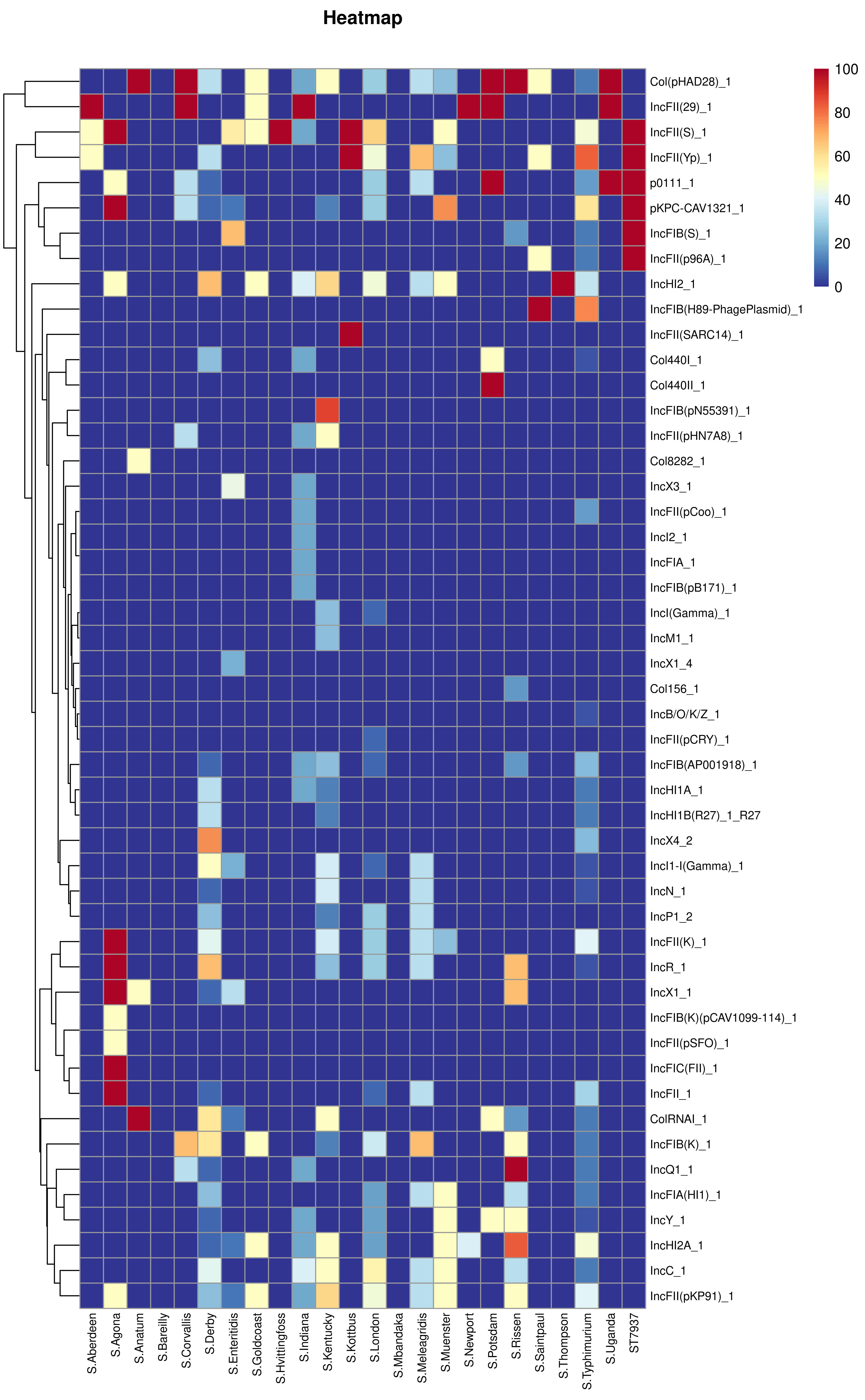

Supplement: Supplementary file 1 [file Data_Sheet_1.ZIP › Fig5_plasmids_different Slamonella strains.tif]

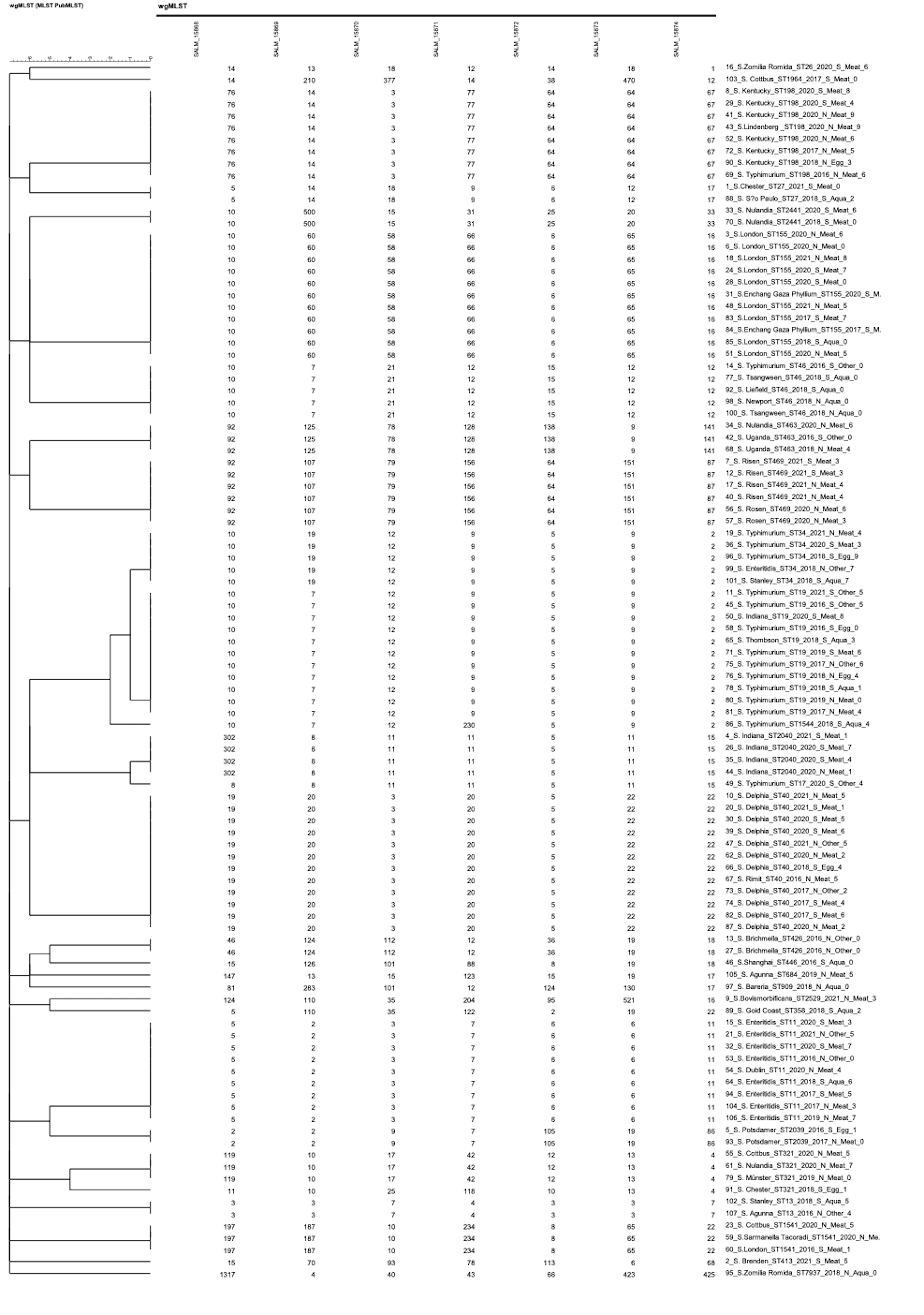

Supplement: Supplementary file 1 [file Data_Sheet_1.ZIP › Fig6_MLST.tif]
